# *RabGAP22* Is Required for Defense to the Vascular Pathogen *Verticillium longisporum* and Contributes to Stomata Immunity

**DOI:** 10.1371/journal.pone.0088187

**Published:** 2014-02-04

**Authors:** Jonas Roos, Sarosh Bejai, Shinichi Oide, Christina Dixelius

**Affiliations:** 1 Department of Plant Biology, Swedish University of Agricultural Sciences, Linnean Center for Plant Biology, Uppsala, Sweden; 2 Molecular Microbiology and Biotechnology group, Research Institute of Innovative Technology for the Earth, Kizugawa, Kyoto, Japan; Agriculture and Agri-Food Canada, Canada

## Abstract

*Verticillium longisporum* is a soil-borne pathogen with a preference for plants within the family *Brassicaceae*. Following invasion of the roots, the fungus proliferates in the plant vascular system leading to stunted plant growth, chlorosis and premature senescence. RabGTPases have been demonstrated to play a crucial role in regulating multiple responses in plants. Here, we report on the identification and characterization of the Rab GTPase-activating protein *RabGAP22* gene from Arabidopsis, as an activator of multiple components in the immune responses to *V. longisporum*. *RabGAP22_Pro_*:*GUS* transgenic lines showed *GUS* expression predominantly in root meristems, vascular tissues and stomata, whereas the RabGAP22 protein localized in the nucleus. Reduced *RabGAP22* transcript levels in mutants of the brassinolide (BL) signaling gene *BRI1-ASSOCIATED RECEPTOR KINASE 1*, together with a reduction of fungal proliferation following BL pretreatment, suggested RabGAP22 to be involved in BL-mediated responses. Pull-down assays revealed SERINE:GLYOXYLATE AMINOTRANSFERASE (AGT1) as an interacting partner during *V. longisporum* infection and bimolecular fluorescence complementation (BiFC) showed the RabGAP22-AGT1 protein complex to be localized in the peroxisomes. Further, fungal-induced *RabGAP22* expression was found to be associated with elevated endogenous levels of the plant hormones jasmonic acid (JA) and abscisic acid (ABA). An inadequate ABA response in *rabgap22-1* mutants, coupled with a stomata-localized expression of *RabGAP22* and impairment of guard cell closure in response to *V. longisporum* and *Pseudomonas syringae*, suggest that *RabGAP22* has multiple roles in innate immunity.

## Introduction

Soil is a complex matrix that hosts a rich diversity of organisms. In the soil, plant roots must compete with other root systems of neighboring plant species for space, water, mineral nutrients, and with the excess of soil organisms feeding on an abundant source of organic material. In the microenvironment surrounding a plant root, the rhizosphere, some soil microorganisms take the advantage of root exudates to invade the root tissue. At the same time the plant immune system is activated upon perception of microbial-associated molecular patterns (MAMPs). It has been described how Arabidopsis roots respond to three selected MAMPs in a highly orchestrated and tissue-specific manner [1]. Still we have limited knowledge on MAMP signaling incited by saprophytes, deleterious soil-borne pathogens and beneficial soil organisms, required to suppress plant immunity pathways upon close interaction and invasion.

A specific host-microbe interaction involves recognition of the foreign organism by the host immune system, leading to amplification of immune responses and subsequent attenuation or elimination of the pathogen. The defense mechanisms include pattern recognition receptor proteins (PRRs), which recognize conserved pathogen-associated molecular patterns (PAMPs) or MAMPs [2]. Activation of PRRs leads to PAMP or MAMP triggered immunity (PTI/MTI), referred to in plants as basal or non-host resistance [3]. A second layer of plant defense consists of recognition of pathogen effector molecules by plant intracellular receptors, the effector-triggered immunity (ETI). In addition, the plant surveillance system is further shaped by a plethora of developmental, hormonal and specific signaling cascades, as well as epigenic components [4], [5], [6], [7], [8]. In all of these processes the cellular machinery plays important roles in receiving and executing proper responses, for example by the vesicle-associated and SNARE protein-mediated exocytosis pathways [9]. The opposite process, endocytosis, is also of importance, exemplified by ligand-triggered internalization of the flagellin-recognition receptor FLS2 [10] and the ethylene-inducing xylanase receptor LeEix2 [11].

The fungal genus *Verticillium* harbors many soil-borne plant pathogens, among them *V. longisporum* that incites disease on several plant species particularly within the family *Brassicaceae* [12], [13]. Microsclerotia, the long-lived soil-infesting resting structures of this hemibiotrophic pathogen, germinate when exposed to root exudates from a host plant. Hyphal growth around lateral roots is followed by direct penetration of the epidermal cells, and subsequent colonization of the xylem vessels [14], [15]. Individual vessels can be filled with growing mycelia whereas others remain normal resulting in vague disease symptoms, particularly at an early disease stage. In the later stages of infection, symptoms including chlorosis, stunting and premature senescence emerge [16]. Formation of microsclerotia in the dying foliage then completes the disease cycle [14], [17]. Knowledge on the plant immune responses taking place during fungal colonization and growth is fragmentary but is gradually expanding. A *Verticillium*-induced transdifferentiation of bundle sheath cells to functional xylem elements suggests that a tissue-specific developmental reprogramming occurs during the infection process [18].

The aim of this study was to increase the knowledge on the components involved in the defense against *V. longisporum.* The starting point chosen was the phenotypic differences in response to *V. longisporum* earlier found in *Brassica oleracea* germplasm [19]. We reasoned that the findings from *B. oleracea* could be exploited in the Arabidopsis system to expand our basic understanding of this particular plant-pathogen interaction. Using a cDNA-AFLP approach, we identified a member in the RabGTPase activating gene family, *RabGAP22,* of importance for plant immunity. RabGTPases make up a large family in Arabidopsis, and the 57 members take part in mechanisms underlying intercellular membrane trafficking, hormone signaling, and stress responses [20], [21], [22], [23]. The GTPases cycle between active GTP-bound, and inactive GDP-bound states. The inherent hydrolysis of GTP to GDP by individual GTPases is a slow process, but is increased by several orders of magnitude by GTPase activating proteins (GAPs). Subsequently, GDP-GTP exchange factors (GEFs) replace the hydrolyzed GDP to GTP, completing the cycle. A family of 24 *RabGAP* genes is present in Arabidopsis [24]. Many eukaryotic RabGAPs contain a Tre-2/Bub2/Cdc16 (TBC) domain architecture. In a comparative evolutionary scan of eukaryotic organism groups using TBC sequences, plant RabGAPs are classified into the TBC-B domain group [25], comprising the Arabidopsis class II subfamily in which RabGAP22 is clustered along with RabGAP11, 19 and 20 [24]. The closest orthologs of RabGAP22 are TBC1D25 in mouse and TBC1D14 in human, the latter being involved in vesicle transport leading to starvation-induced autophagy by interactions with the autophagy kinase ULK1 [26]. In the present study we found an involvement of RabGAP22 in jasmonic acid (JA) accumulation and JA and brassinolide (BL) signaling. We also uncovered a role for RabGAP22 in stomatal responses against both *V. longisporum* and *Pseudomonas syringae* DC3000. Together, our data revealed RabGAP22 to be a mediator of multiple responses in plant immunity.

## Results

### *RabGAP22* is Required for Immunity to *V. Longisporum*

**Figure 1:**
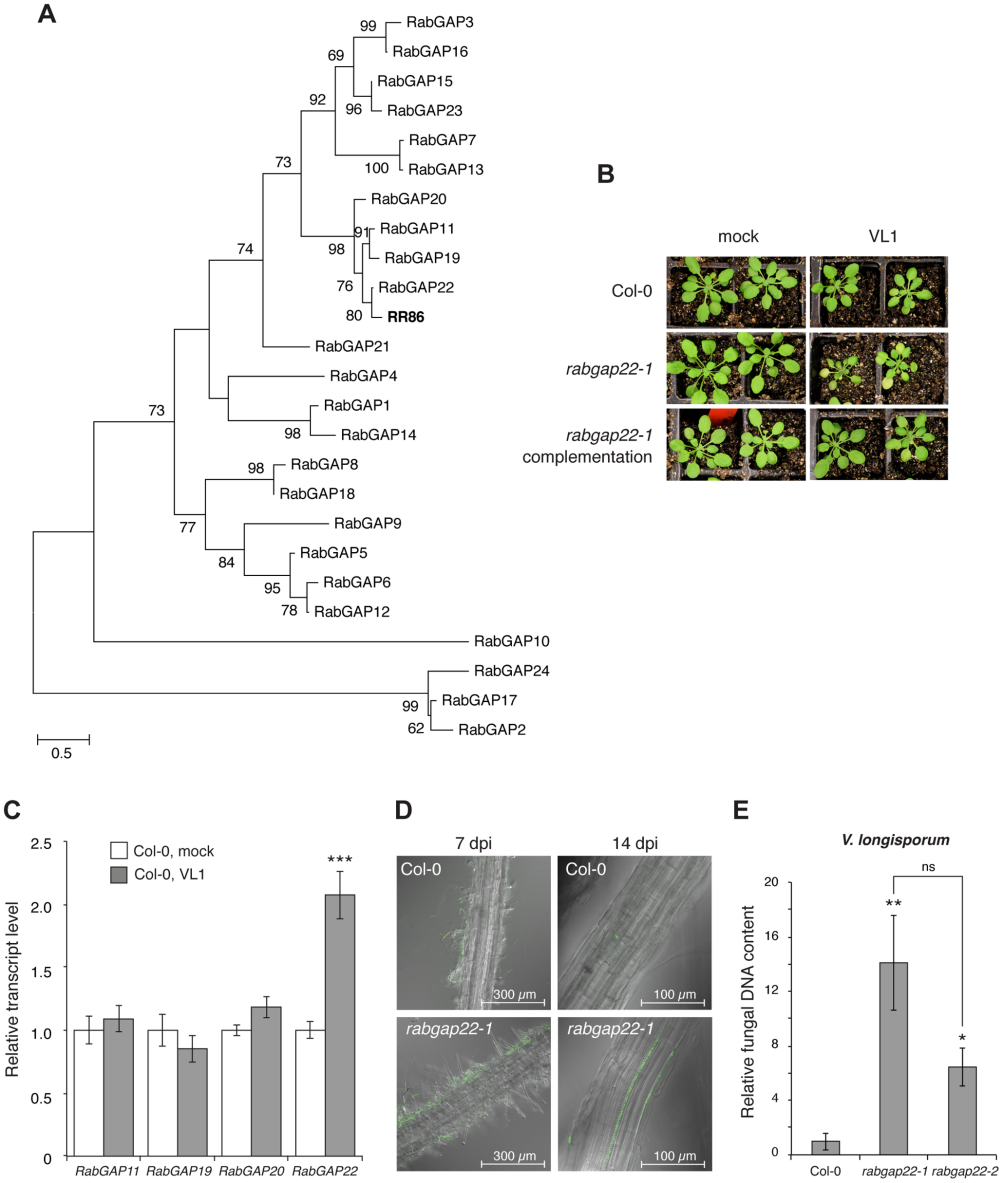
Arabidopsis *RabGAP22* is required for defense to *V. longisporum*. (A) The transcript-derived fragment RR86 from *Brassica oleracea* accession BRA723 cluster with four Arabidopsis *RabGAP* genes in maximum likelihood analysis. Bootstrap values >50 are shown. Scale bar represents the number of substitutions per site. (B) Phenotypes of soil-grown plants 28 days post inoculation with *V. longisporum* showing strong symptoms in *rabgap22-1* plants (chlorosis, stunting and premature senescence) and only mild symptoms in Col-0. Complementation with the native gene (*RabGAP22_Pro_:RabGAP22*) restored *rabgap22-1* to the wild-type phenotype. (C) Relative transcript levels of *RabGAP11*, *RabGAP19*, *RabGAP20* and *RabGAP22* in roots of *in vitro*-grown Col-0 at 2 d post inoculation with *V. longisporum*. Data represent means ± SE (n = three pools of >20 plants, repeated twice). (D) Fungal colonization in roots of plants grown in hydroponic culture. Images taken at 7 and 14 d post inoculation with GFP-tagged *V. longisporum*. (E) Fungal DNA content in roots of plants grown in hydroponic culture at 14 dpi, quantified with qRT-PCR. Data represent means ± SE (n = 3 pools of 5 plants). Asterisk indicates significant difference to Col-0. (Student’s t-test; *p≤0.05; **p≤0.01; ***p≤0.001; ns = not significant).

The white cabbage accession line BRA723 was previously identified as highly resistant to *V. longisporum* infection [19]. Hence, this material was selected for a cDNA-AFLP approach to further explore defense responses in this plant-pathogen system. A number of differentially expressed transcripts in roots during fungal interaction were detected. At two days post inoculation (dpi), 104 transcript-derived fragments were isolated and sequenced (Table S1). When comparing the sequences with the Arabidopsis database, 32 of the differentially expressed genes were identified as potential defense gene candidates. Out of these, the most strongly up-regulated transcript, RR86 (Accession number: KF258677), was further examined. BLASTP analysis identified four Arabidopsis genes homologous to RR86: At3g49350 (E = 3e^−1^), At5g24390 (E = 2e^−05^), At5g41940 (E = 2e^−05^) and At5g53570 (E = 5e^−52^), corresponding to the earlier classified *RabGAP11*, *19, 20* and *22,* respectively [24]. Phylogenetic analysis on amino acid sequences from all 24 Arabidopsis RabGAPs and RR86 also placed RR86 in the same cluster as these four *RabGAP* genes (Figure 1A).

To determine if any of the four *RabGAP* gene candidates were linked to plant immunity, their corresponding Arabidopsis T-DNA insertion mutants (Figure S1A) were investigated. None of these showed any difference in developmental phenotype compared to wild-type Col-0. When screened for responses to *V. longisporum*, the homozygous *rabgap22-1* plants showed a clear susceptible phenotype (Figure 1B), whereas the *rabgap11-1*, *rabgap19-1* and *rabgap20-1* mutants displayed responses similar to that of wild-type (Figure S1b). To validate theses phenotypic responses quantitative real-time PCR (qRT-PCR) analyzes were performed. All T-DNA insertion mutant plants were found to be significantly down-regulated in their target genes (Figure S1C), and only the *RabGAP22* gene was triggered by the fungus in wild-type Col-0 (Figure 1C). These results, together with complementation analysis using a *RabGAP22_Pro_:RabGAP22* construct that restored the susceptible *rabgap22-1* phenotype to the resistant wild-type phenotype (Figure 1B), demonstrated the importance of *RabGAP22* in defense to *V. longisporum*. Fungal growth in the plant roots was also monitored using *GFP*-tagged *V. longisporum*. At 14 dpi, the fungus was detected in roots of both Col-0 and mutant plants. In agreement with the earlier results the degree of colonization was found to be significantly higher in *rabgap22-1* and *rabgap22-1* plants (Figures 1D, E).

### *RabGAP22* is Expressed in Vascular Tissues and Stomatal Guard Cells

**Figure 2:**
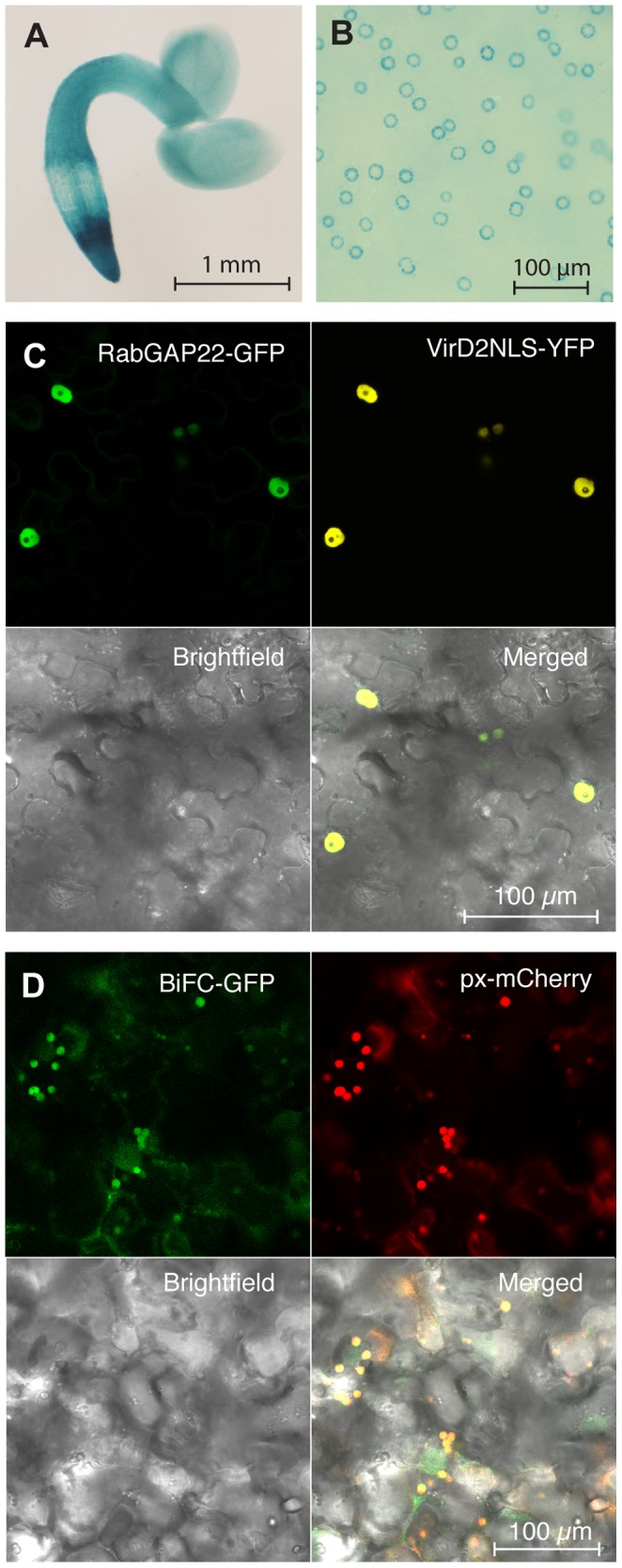
*RabGAP22* is expressed in stomata and the protein localizes in the nucleus. Histochemical localization of GUS activity in *in vitro*-grown transgenic Arabidopsis Col-0 plants harboring a *RabGAP22_Pro_*:*GUS* construct. (A) 2 days-old seedling with staining in the entire plant, in particular in root meristems. (B) Leaf from 7 days-old seedling, with GUS staining in stomatal guard cells. (C, D) *Agrobacterium*-mediated co-infiltration of transgenic constructs in *Nicotiana benthamiana* leaves. (C) Co-infiltration of a *RabGAP22_Pro_*:*RabGAP22-GFP* construct and the nuclear reference marker VirD2NLS-YFP. Both constructs show localization in the nucleus. (D) Bimolecular Fluorescence Complementation (BiFC) analysis using *pSITE-cEGFP-AGT1* and *pSITE-nEGFP-RabGAP22* plasmids. Reconstituted GFP signal was detected in the peroxisomes and was verified with a peroxisome-targeted mCherry marker (px-mCherry). Confocal images were taken 4 days post inoculation, using a Zeiss 780 confocal scanning microscope.

In order to monitor the tissue and organ-specific expression of *RabGAP22,* transgenic Col-0 plants harboring a *RabGAP22_Pro_*:*GUS* construct were made. GUS-staining revealed expression throughout the plant, most strongly in root meristems, vascular tissues, stomata, trichomes and flower tissues such as style and receptacle (Figures 2A, B and S2A–G). This gene expression pattern was only very subtly changed at 2 and 6 days post *V. longisporum* inoculation (data not shown). We further searched for the subcellular localization of RabGAP22. No information could be acquired by TargetP [27] but the search tools MultiLoc2 [28] and AtSubP [29] suggested a nuclear localization. To clarify this, *Nicotiana benthamiana* plants were infiltrated with a *RabGAP22_Pro_:RabGAP22-GFP* construct, which demonstrated a nuclear localization of the RabGAP22-GFP protein (Figure 2C). This observation was further supported by co-infiltration of *N. benthamiana* leaves with *RabGAP22_Pro_*:*RabGAP22*-*GFP* and the nuclear marker VirD2NLS-YFP construct, showing a clear co-localization of the GFP and YFP signals in the nucleus (Figure 2C).

### RabGAP22 Interacts with the Photorespiratory Protein AGT1

To gain further insight into the function of RabGAP22 in plant immunity, we looked for potential interacting proteins under stress conditions. Transgenic Arabidopsis plants harboring *35S_Pro_:RabGAP22-*His were made, and immunoprecipitation experiments with anti-His antibodies were performed. In leaves from mock-treated plants, only the ∼60 kDa band corresponding to the RabGAP22-His protein was observed, whereas in leaf samples from *V. longisporum* soil-inoculated plants, a protein of ∼45 kDa co-precipitated with the RabGAP22-His protein (Figure S3A). The amino acid sequence of this new protein (Accession number: KF242188) was identified by MALDI-MS/MS as SERINE:GLYOXYLATE AMINOTRANSFERASE 1 (AGT1, At2g13360) (Figure S3B), an enzyme in the photorespiratory pathway [30]. An *AGT1* homolog is known to be indispensable for appressorium function in the *Magnaporthe oryzae* fungus [31], thus we found it important to clarify if the identified AGT1 protein was of plant or fungal origin. When AGT1 protein sequences from *V. longisporum, M. oryzae* and Arabidopsis were aligned, the protein fragments from MALDI-MS/MS showed a perfect match to Arabidopsis AGT1 verifying a plant origin (Figure S3C). qRT-PCR analysis also revealed an increase in *AGT1* transcript levels in roots of *V. longisporum* inoculated plants at 2 dpi (Figure S3D). To generate more data along this line, a bimolecular fluorescence complementation (BiFC) assay [32] was performed to show if interaction of the two proteins occurs *in planta*. Two plasmids, in which RabGAP22 and AGT1 were fused to the GFP C- and N-termini respectively (*pSITE-cEGFP-RabGAP22* and *pSITE-nEGFP-AGT1*) were constructed and infiltrated into *N. benthamiana* leaves. A clear fluorescence signal was detected in the peroxisomes, demonstrating interaction of the two proteins (Figure 2D). The peroxisomal localization was further supported by co-infiltrations of the two BiFC constructs and a mCherry-tagged peroxisomal marker (Figure 2D). As a control step, the *pSITE-cEGFP-RabGAP22* and *pSITE-nEGFP-AGT1* plasmids were infiltrated individually into tobacco leaves to check for potential interactions, but no fluorescence signals were detected from those samples. The observed peroxisomal localization of RabGAP22-AGT1 was not unlikely, as AGT1 is a peroxisome-localized protein [33]. Presence of RabGAP22 in both nuclear and peroxisomal cellular compartments is also in line with the role of RabGAP proteins in vesicle trafficking between membrane compartments [34]. When tested for the response to *V. longisporum*, *agt1* T-DNA insertions mutants showed a phenotype indistinguishable to that of Col-0 (Figure S3E), indicating that the immune response is mainly mediated by RabGAP22. The lack of a peroxisomal localization of RabGAP22-GFP in unstressed plants (Figure 2C) suggests that a specific re-localization of RabGAP22 to peroxisomes takes place under biotic stress conditions, here exemplified in response to *V. longisporum*. Changes in subcellular localization and activity of GTPases, GAPs and GEFs frequently occur in response to changes in the GTP/GDP-bound state [35] and also in response to phosphorylation of individual residues in these molecules [36]. The rice Rab protein OsRab11 is characterized both for regulating vesicular trafficking between the trans-Golgi network and vacuole [37], [38] and for interaction with the peroxisomal protein OsOPR8 [39].

Peroxisomal proteins are involved in a number of processes associated to pathogen defense responses, not least H_2_O_2_-production and jasmonic acid (JA) biosynthesis, reviewed by Sørhagen *et al*. [40]. Consequently we assayed these responses in *rabgap22-1* plants, starting with measurement of H_2_O_2_ levels by 3,3′-diaminobenzidine (DAB) staining. The basal level of H_2_O_2_ was lower in the mutant compared to Col-0, and in contrast to the wild-type, no increase in the levels were detected in response to *V. longisporum* (Figures S3F, G). We interpreted the data as a very modest involvement of RabGAP22 in the H_2_O_2_ response, and instead initiated analysis of JA biosynthesis and signaling.

### *V. Longisporum* Promotes Increased JA and JA-Ile Levels

**Figure 3:**
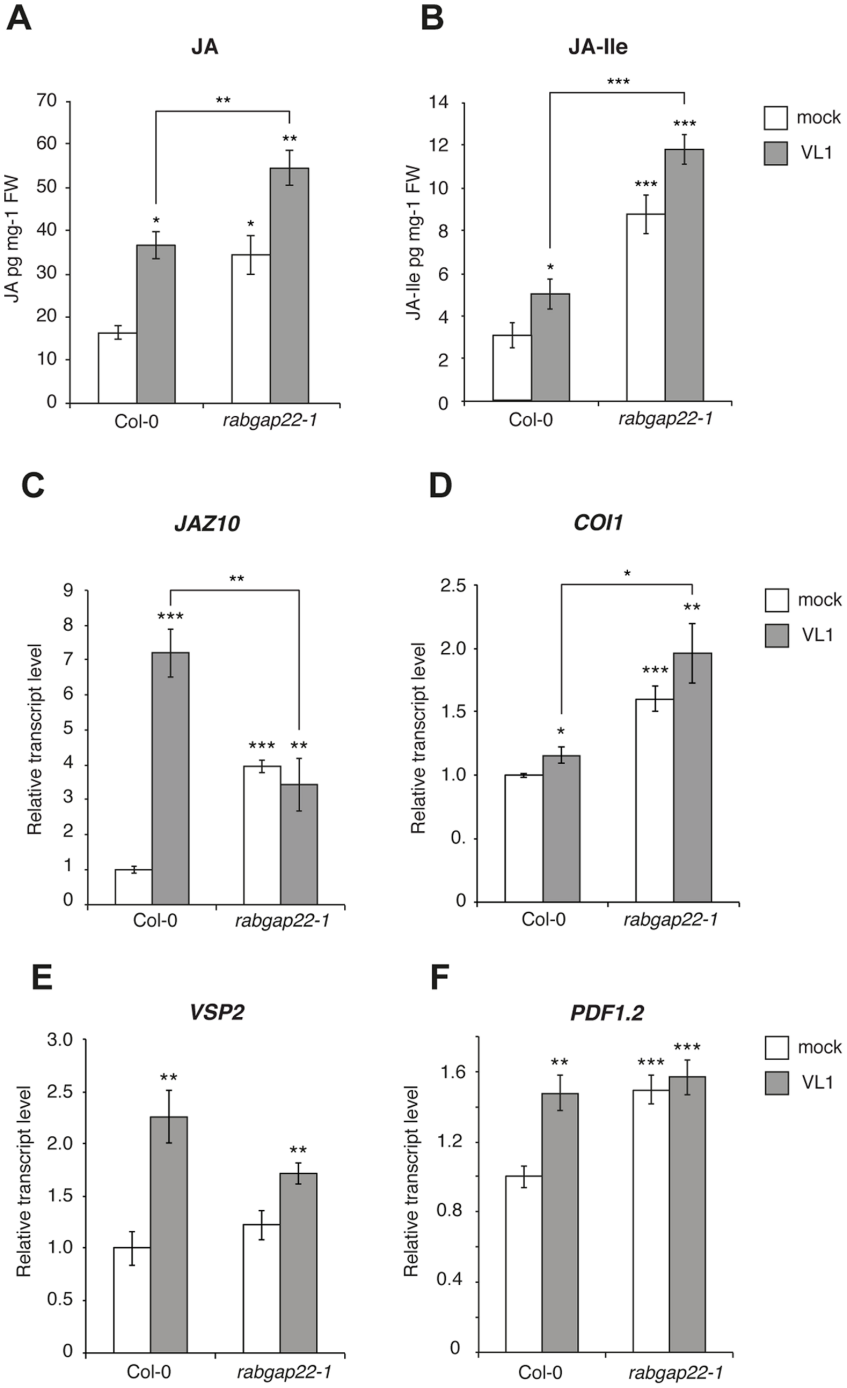
*RabGAP22* represses jasmonic acid (JA) levels and JA signaling. (A, B) Endogenous hormone content in *in vitro*-grown Arabidopsis wild-type and *rabgap22-1* plants 2 d post inoculation with *V. longisporum*. (A) JA and (B) JA-Isoleucine (JA-Ile). Data represent means ± SE (n = 3 pools of >50 plants). (C–F) Relative transcript levels of the JA signaling components (C) *JAZ10,* (D) *COI1,* (E) *VSP2* and (F) *PDF1.2* in roots of *in vitro*-grown Arabidopsis plants 2 d post inoculation with *V. longisporum*. Data represent means ± SE (n = 3 pools of >20 plants, repeated twice). Asterisks indicate significant difference to the respective Col-0 mock treated control (Student’s t-test; *p≤0.05; **p≤0.01; ***p≤0.001).

Jasmonic acid (JA) and JA signaling play important roles in most plant defense systems, in particular in responses to necrotrophic and hemibiotrophic pathogens. To elucidate if the increased susceptibility of *rabgap22-1* was linked to alterations in JA-mediated defense responses, endogenous levels of both JA and the bioactive JA-Isoleucine (JA-Ile) were measured at 2 dpi. As expected, both compounds increased in *V. longisporum* challenged Col-0 plants (Figure 3A, B). However, the levels of JA and JA-Ile were significantly higher in the *rabgap22-1* mutant and strikingly induced by the fungus, indicating an important role of RabGAP22 in the JA biosynthesis pathway. In order to understand more on JA-associated events we analyzed transcript levels of key genes in the JA signaling pathway. The basal transcript levels of the repressor of JA-signaling *JASMONATE ZIM-DOMAIN 10* (*JAZ10*) were 4-fold higher in *rabgap22-1* compared to wild-type, but did not change upon fungal challenge (Figure 3C). Whereas levels of the JA signaling marker *CORONATINE INSENSITIVE 1* (*COI1*) only increased slightly in Col-0 in response to *V. longisporum*, elevated levels were seen in both mock and inoculated *rabgap22-1* plants (Figure 3D). In agreement with previous reports [17], both the JA marker *VEGETATIVE STORAGE PROTEIN 2* (*VSP2*) and the JA/ethylene marker *PLANT DEFENSIN 1.2* (*PDF1.2*) were induced by *V. longisporum* in the wild-type (Figures 3E, F). In contrast, the induced levels of *VSP2* in *rabgap22-1* were lower compared to Col-0, whereas *PDF1.2* levels appeared constitutively high in the mutant. Overall, our results indicate that RabGAP22 is a negative regulator of JA responses and that over-production of JA and JA-Ile in the *rabgap22-1* mutant is accentuated during *V. longisporum* infection, contributing to a distorted JA defense signaling and an enhanced susceptible phenotype.

### RabGAP22 is Involved in Brassinosteroid-associated Responses

**Figure 4:**
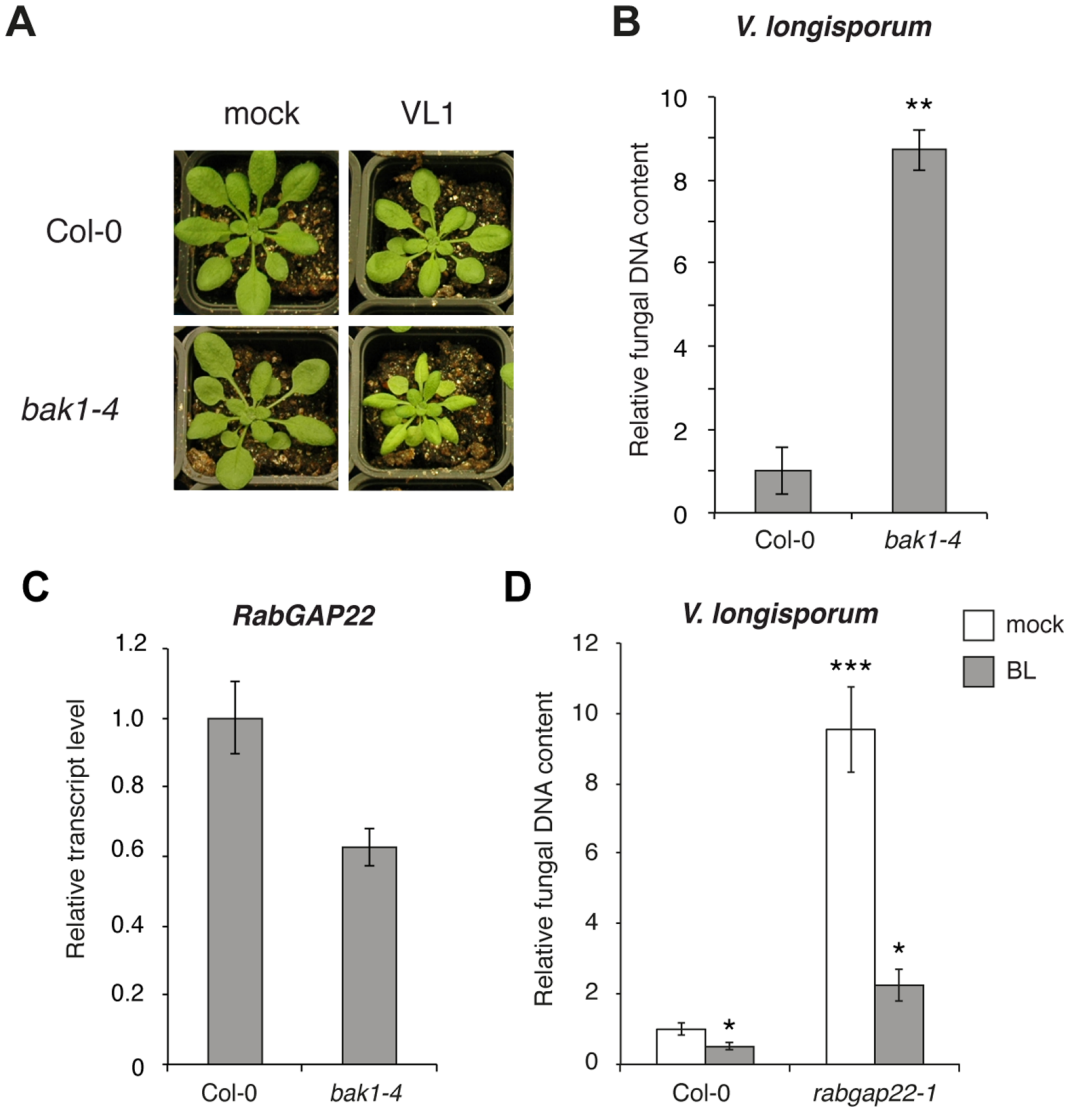
BAK1 and brassinolide signaling are required for RabGAP22-mediated responses. (A) Phenotypes of soil-grown plants 21 days post inoculation with *V. longisporum.* Whereas symptoms in wild-type Col-0 are mild, *bak1–4* plants show strong symptoms (chlorosis, stunting and premature senescence) of *V. longisporum* infection. (B) Fungal DNA content in roots of plants grown in hydroponic culture, quantified at 14 dpi using qRT-PCR. Data represent means ± SE (n = 3 pools of 5 plants). (C) Relative transcript levels of *RabGAP22* in roots of two-week-old *in vitro* grown Col-0 and *bak1–4* mutants. (D) Fungal DNA content in roots of plants grown in hydroponic culture at 14 dpi, quantified with qRT-PCR. Prior to fungal inoculation, plants were treated with either water or 24-epibrassinolide (BL) for two days. Data represent means ± SE (n = 3 pools of 5 plants). Asterisk indicates significant difference to Col-0. (Student’s t-test; *p≤0.05; **p≤0.01; ***p≤0.001).

To further expand our understanding of *RabGAP22* defense responses, we searched for co-expressed genes. A comparative analysis of all available microarray datasets deposited in Genevestigator [41] was performed. The similarity search tool limited to perturbations for *RabGAP22* showed *MITOGEN-ACTIVATED PROTEIN KINASE PHOSPHATASE* (*MPK1*) and *BRI1-ASSOCIATED RECEPTOR KINASE 1* (*BAK1*) to be the two most correlated genes with a score of 0.59 and 0.56, respectively (Figure S4A). BAK1 is known to affect multiple processes, including brassinosteroid signaling and defense to plant pathogens [42], [43], [44]. Hence the *bak1–4* Arabidopsis null mutant was challenged with *V. longisporum* to investigate its potential contribution in this pathosystem. The *bak1–4* plants displayed a clear susceptible phenotype as well as a massive fungal colonization at 14 dpi compared to the wild-type (Figures 4A, B). In addition, *BAK1_Pro_:GUS* transgenic Arabidopsis plants displayed elevated levels of GUS expression in response to *V. longisporum* at 1 and 2 dpi (Figure S5). Further, the finding of attenuated levels of *RabGAP22* in the *bak1–4* mutant (Figure 4C) indicates that *BAK1* may be required for *RabGAP22* function.

When our Genevestigator search was limited to hormone responses, brassinolide (BL) treatment was found to induce the highest *RabGAP22* expression (Figure S4B). Brassinosteroids (BRs) are known to modulate plant defense responses and are involved in many other developmental processes including xylem differentiation [45], [46]. We therefore analyzed the responses to 24-epibrassinolide (BL) in our plant materials. No difference in the BL-induced root growth inhibition was found in *rabgap22-1* compared to Col-0 plants grown *in vitro*, revealing that *rabgap22-1* mutants were not compromised in the uptake of BL (data not shown). However when we pretreated plants with BL prior to *V. longisporum* inoculation, fungal colonization was reduced 4-fold in *rabgap22-1* and 2-fold in wild-type (Figure 4D). Together these results suggest that the increased fungal colonization in *rabgap22-1* is in part due to impaired BL signaling, and that RabGAP22 is a downstream component of BL-mediated signaling.

### *RabGAP22* Affects Stomata Closure through Attenuated ABA Levels

**Figure 5:**
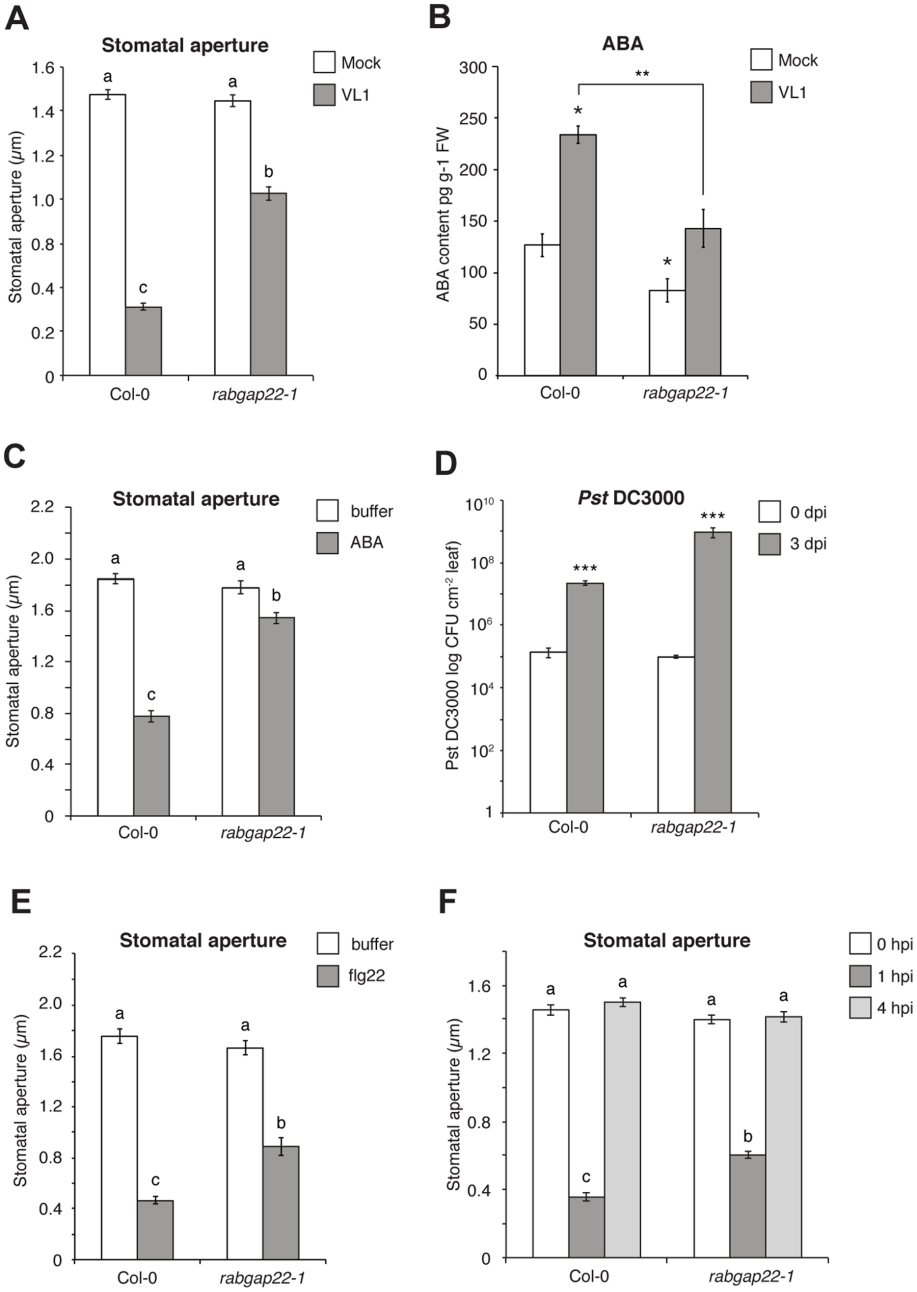
*RabGAP22* contributes to stomatal immunity. (A) Stomatal apertures in soil-grown mock and *V. longisporum* inoculated plants at 14 dpi. Data represent means ± SE (>60 randomly selected stomata). (B) Endogenous abscisic acid (ABA) content in *in vitro*-grown Arabidopsis wild-type and *rabgap22-1* plants 2 d post inoculation (dpi) with *V. longisporum*. Data represent means ± SE (n = 3 pools of >50 plants). (C) Stomatal apertures in four-week-old soil-grown plants, in response to ABA treatment. Data represent means ± SE (>60 randomly selected stomata). (D) Quantification of bacterial growth in soil-grown Arabidopsis plants 0 and 3 days post spray inoculation with *Pst* DC3000. Average log colony forming units (CFU) per cm^2^ leaf area are shown. Data represent means ± SD (n = 5 pools of 3 leaves). (E, F) Stomatal apertures in four-week-old soil-grown plants, in response to (E) flg22 treatment and (F) *Pseudomonas syringae* DC3000 spray inoculation. Data represent means ± SE (>60 randomly selected stomata). Asterisks indicate significant difference to the respective mock treated control. (Student’s t-test; *p≤0.05, **p≤0.01, ***p≤0.001). Different letters indicate significant difference (one-way ANOVA followed by a Tukey HSD test, 95% confidence interval).

The observed *RabGAP22_Pro_:GUS* expression in guard cells (Figure 2B) resembles that of Arabidopsis ROP11 GTPase [47], a negative regulator of ABA-mediated stomatal closure. ABA is a central regulator of the stomatal apparatus [48], and Genevestigator expression data suggested a reduction in *RabGAP22* expression in response to ABA treatment (Figure S4B). These features prompted us to test the behavior of stomata in response to *V. longisporum* challenge. At 14 dpi we found stomatal apertures in wild-type Col-0 to be reduced by 75% compared to mock treated plants. A feature that could explain the increased drought tolerance of *V. longisporum* inoculated Col-0 seen at a similar time-point [18]. At 14 dpi stomata were in contrast partially open (reduced by 34%) in the *rabgap22-1* plants upon pathogen attack (Figure 5A). We wanted to know if this impairment in stomata closure could be linked to altered endogenous ABA levels in our plant materials. Congruent with previous findings [17] we detected a significant increase in ABA content in leaves of Col-0 in response to *V. longisporum* (Figure 5B), whereas the induction of ABA in the *rabgap22-1* mutant only reached half the levels compared to the wild-type. These results were followed by a closer monitoring of the stomatal apparatus in Col-0 and *rabgap22-1* plants in response to ABA treatment. No anomalies in size and numbers were found but stomata in *rabgap22-1* were impaired in ABA-induced closure, with stomatal apertures only being reduced by 14% in response to the hormone application (Figure 5C). Based on the partial impaired stomatal closure in *rabgap22-1* in response to *V. longisporum*, experiments were also run to clarify if a similar influence on the stomata apparatus could be seen in the well-studied *Pseudomonas syringae* DC3000 (*Pst* DC3000) system. When inoculated with *Pst* DC3000, leaves of *rabgap22-1* plants showed a strong increase in bacterial proliferation compared to wild-type at 3 dpi (Figure 5D). PAMPs from *Pst* DC3000 are known to trigger stomatal closure in Arabidopsis within 1 to 2 h post inoculation through ABA biosynthesis and signaling [49], [50], [51], as well as via an ABA-independent oxylipin pathway [52]. The bacterial effector coronatine (COR) inhibits this effect by reopening the stomata within 3 to 4 h after infection. Treatment with flg22 (a PAMP of *Pst* DC3000) on our materials triggered stomatal closure in wild-type Col-0 plants as expected but this response was partially impaired in *rabgap22-1* (Figure 5E). Stomatal apertures were also measured in *Pst* DC3000 inoculated plants, revealing at 1 hpi a weak, but significant reduction in the stomatal closure response of the *rabgap22-1* plants (Figure 5F). At 4 hpi, stomatal apertures in both Col-0 and *rabgap22-1* were again open indicating that COR-mediated re-opening of stomata was not effected in the mutant. That the stomatal closure responses to flg22 and *Pst* DC3000 were not as strong as those for *V. longisporum*, could be attributed to involvement of additional defense components in the case of this leaf pathogen.

## Discussion

Soil-borne fungal pathogens are causal agents of diseases of increasing economic importance. In comparison to plant responses to foliar pathogens, relatively little is known about responses to root infecting pathogens primarily due to the difficulty in observing the early stages of the interaction. At present only a few important components in the plant immune response pathways to *Verticillium* fungi are known. Using a positional cloning strategy, the *Ve1* and *Ve2* genes conferring resistance to *V. dahliae* and *V. albo-atrum* were cloned in tomato [53]. Both *Ve1* and *Ve2* genes encode cell surface receptors with an extracellular leucine-rich repeat (LRR) domain but are lacking a cytoplasmic signaling domain, characteristic for receptor-like proteins [54]. The responses of the *Ve* genes have also been evaluated in transgenic Arabidopsis because no closely related *Ve* orthologs are present it its genome. *Ve1* in the Arabidopsis genomic background conferred resistance to *V. dahliae* but not to *V. longisporum* [55] suggesting that another PRR is operating in the latter case. An Arabidopsis mutant in a cell wall-associated member of the receptor-like kinase (RLK) gene family mediating resistance to *Fusarium oxysporum* (*rfo1*) was earlier shown to promote susceptibility to *V. longisporum* infection [12], and could be indicative of important groups of RLKs to be further examined.

The fungal avirulence gene *Ave1*, a natriuretic peptide, is present in several fungal species including *V. dahliae* and is a proposed interactor to *Ve1* [56]. We queried our *V. longisporum* genome sequences for the presence of *Ave1* orthologs but no similar sequences could be found. In our efforts to find a candidate effector capable of binding to RabGAP22, we extended the searches to include a wider group of organisms. Effectors for example from the intracellular *Shigella flexneri* bacteria exhibit RabGAP activity [22]. Via GTP hydrolysis of the host RabGTPase Rab1, VirA disrupts ER-to-Golgi trafficking, allowing the bacteria to escape the autophagy-mediated defense. In common with other RabGAPs, VirA contains a TBC domain. Hence, we used TBC sequences retrieved from various organisms in the search for orthologs in the *V. dahliae* genome [57], and in our genomic sequences from *V. longisporum,* without being able to identify an obvious effector candidate of this nature.

Members of the somatic embryogenesis receptor-like protein kinase (SERK) gene family in Arabidopsis, *SERK1* and *SERK4* together with *SERK3*, also known as *BAK1,* are required for *Ve*-mediated defense against *V. dahliae* [55]. Here we revealed BAK1 to be an important defense component also to *V. longisporum* contributing to *RabGAP22* expression. It could be speculated whether RabGAP22 functions together with BAK1 in a network similar to that in the *Magnaporthae oryzae*/chitin- rice interaction. In that system the small GTPase OsRac1 is an essential component of a receptor complex in chitin-induced immunity, together with OsRacGEF1, chitin-binding OSCEBiP and the receptor-like kinase OsCERK1 [58]. As the BR receptors BAK1 and BRASSINOSTEROID INSENSITIVE 1 (BRI1) constantly recycle via endosomes [59], RabGAP22 could also take part in BAK1 recycling, thereby indirectly regulating appropriate BL or PAMP responses. In the present study, BL treatment led to a significant reduction in disease susceptibility towards *V. longisporum* in both wild-type Col-0 and the *rabgap22-1* mutant. A similar induced resistance response was found on tomato inoculated with *V. dahliae* after an exogenous application of BR [60]. This type of BR-enhanced resistance has been suggested to be a result of cross-talk with other plant hormones, and thus different from the BAK1-mediated responses.

The formation of an AGT1-RabGAP22 complex characteristic for *V. longisporum* challenged plants suggests an activation of peroxisomal processes by RabGAP22, in line with the emerging role of peroxisome-produced H_2_O_2_ in pathogen defense responses [61], [62]. Proteomic analysis of the xylem sap from *V. longisporum* inoculated *B. napus* plants identified fungal genes involved in oxidative stress, whereof a catalase peroxidase was found to play a key role for the late infection phase [63]. We observed however only a mild attenuation of H_2_O_2_ production in the susceptible *rabgap22-1* plants, indicating that H_2_O_2_ is not a main component of the RabGAP22-mediated defense.

Jasmonic acid is synthesized through the octadecanoid pathway that involves the translocation of lipid intermediates from the chloroplast membranes to the cytoplasm and later on into peroxisomes where further processing steps take place to complete the JA biosynthesis, reviewed by Kombrink [64]. JA-Ile, conjugated in the cytoplasm then triggers the JA-dependent signaling cascades. Arabidopsis OPDA REDUCTASE 3 (OPR3) catalyzes the reduction of 12-oxo-phytodienoic acid (OPDA) in the first step of JA biosynthesis taking place in the peroxisomes. In rice, Rab11 interacts with OPDA REDUCTASE 8 (OsOPR8) in the peroxisomes, and presumably this association activates the OPDA-reducing activity of OsOPR8, leading to induced JA-mediated defense signaling [39]. In our case however, RabGAP22 has an inhibitory effect on the JA signaling, possibly via its interaction with peroxisomal AGT1. Modification of OPDA to JA includes several steps, including three rounds of β-oxidation and each of the various enzymes required for the modifications need to be imported into the peroxisome mediated by peroxins (PEXs). The small RabGTPase RabE1c in Arabidopsis has recently been shown to interact with PEX7 on the peroxisomal membrane [23], a process important for peroxisome biogenesis and subsequent β-oxidation. The photorespiratory gene *AGT1* has multiple functions and currently we can only speculate that together with RabGAP22 it interferes with other unidentified factors required for JA biosynthesis. In either case, the accentuated JA signaling in the *rabgap22-1* plants may also impact abscisic acid (ABA) signaling, as extensive crosstalk takes place between the JA and ABA pathways, not least in pathogen responses [4], [65]. In the case of the *rabgap22-1* mutant, the reduced ABA levels indicate that ABA may be negatively impacted by the substantial increases in JA and JA-Ile levels.

Stomata consist of two guard cells that function as turgor-operated valves regulating the gas exchange in plants. The guard cells are also capable of closing in response to PAMPs such as flg22 and lipopolysaccharides from *P. syringae* [49], [50]. flg22-triggered stomatal closure, acting through the induction of SA and ABA biosynthesis and signaling is rapidly inhibited by the bacterial effector COR which re-opens the stomata 3–4 h after infection, through the actions of NAC transcription factors regulated by MYC2 [66], [67]. The partial failure of *rabgap22-1* stomatal closure in response to *V. longisporum* and ABA, but also to some extent to flg22 and *Pst* DC3000, points to a presumed role for RabGAP22 in mediating correct ABA- and stomatal immunity-associated signals. The Arabidopsis GTPase ROP11 negatively regulates stomatal closure, by direct interaction with abscisic acid insensitive 1 (ABI1), a signaling component downstream of the ABA receptor RCAR1/PYL9 [68]. Whether RabGAP22 functions individually or in concert with other GTPases such as ROP11 in regulating this process is currently unknown.

### Conclusions

Evidence of involvement of RabGTPases and RabGAPs in diseases has its basis from human and animal model systems [69], [70], and roles of these molecules in plant pathogen interactions are emerging [21], [22], [23]. Here, we characterize Arabidopsis RabGAP22 as a component required for resistance to *V. longisporum*, and in this early stage we can only speculate on its function in plant immunity (Figure S6). Our results showed a clear interaction of RabGAP22 and AGT1 in the peroxisomes of *V. longisporum*-challenged plants. Together with the increased JA content in *rabgap22-1*, this warrants the hypothesis that RabGAP22-AGT1 may interfere with peroxisomal-localized steps in the JA biosynthesis. The strong reduction in *V. longisporum* colonization in *rabgap22-1* upon BL treatment clearly indicates that BL signaling is important for RabGAP22 function. However the exact involvement of the BL signaling component *BAK1* remains elusive at this stage. We also observed a stomatal closure response in *V. longisporum*-challenged wild-type plants. This response was impaired in *rabgap22-1*, and stomata in the mutant further failed to close in response to ABA, suggesting a role for RabGAP22 in ABA-mediated stomatal closure. The multiple effects of RabGAP22 found here in the Arabidopsis - *V. longisporum* interaction are intriguing. At present we only have a fragmentary knowledge on the role of RabGAP22 in the response to *V. longisporum*, and further efforts remain to gain a full understanding of the multiple processes it is involved in.

## Materials and Methods

### Plant Material

Arabidopsis genotypes (Col-0) (N1092) and T-DNA insertion mutants *rabgap11-1* (SALK_093855), *rabgap19-1* (SALK_114911), *rabgap20-1* (SALK_039920), *rabgap22-1* (SALK_069842), *rabgap22-2* (SALK_073221), *agt1* (SALK_104969) and *bak1–4* (SALK_116202; [42]) were used in the study together with *BAK1_Pro_:GUS* [71] and *Brassica oleracea* cultivar group *capitata,* accession BRA723 [19]. Plants were grown on soil [72] or *in vitro* [73]. For transcription analysis on roots, plants were grown in liquid MS medium to facilitate extractions.

### Pathogens, Plant Inoculation and Pathogen Quantification

The *Verticillium longisporum* isolate CBS110220 (here renamed VL1) [74], [75] was used throughout the study, except for studies of root colonization, where GFP-tagged *V. longisporum* VL 43 [15] was used. For soil infections, roots of two-week-old soil-grown Arabidopsis plants were dipped for 10 min in a 10^7^ conidia ml^−1^ suspension. Inoculated plants were re-potted in sterile soil, humidity raised to 100% for 2 days, and disease progress monitored for 3–4 weeks post inoculation. Plants were scored for disease susceptibility based on the appearance and severity of chlorosis, stunting and premature senescence. Typically, three separate stages of disease were distinguished between: 1 = discoloration of leaf vascular tissues; 2 = stunting and leaf chlorosis being to appear; 3 = severe stunting and chlorosis compared to control. For *in vitro* liquid cultures, two-week-old (Arabidopsis) or 6-days-old (*B. oleracea*) plants were washed with sterile H_2_O and transferred to fresh MS medium without sucrose supplemented with 10^4^ VL1 conidia ml^−1^. Quantification of *V. longisporum* DNA in plants grown in hydroponic culture [55] was carried out 14 d post inoculation. Following a five-minute wash in 70% ethanol, biological replicates (roots from at least five plants) were collected. This method ensured the quantification of fungus inhabiting the vascular tissues. Quantitative real-time PCR (qRT-PCR) pathogen quantification was performed as described below with primers provided in Table S2. *Pseudomonas syringae* pv. tomato DC3000 was maintained at 28°C on King’s B medium supplemented with rifampicin (50 µg ml^−1^). Four-week-old Arabidopsis plants were spray inoculated [76] with a 10^8^ CFU ml^−1^ bacterial suspension and kept under high humidity for disease development. At 3 dpi, biological replicates (at least three leaves) were collected and surface sterilized with ethanol (70% v/v). Samples were ground in sterile water in a TissueLyser (Qiagen, http://www.qiagen.com/), serially diluted, and grown on King’s B medium containing rifampicin (50 µg ml^−1^). Colonies were counted after 40 h.

### Brassinolide Treatment

Quantification of *V. longisporum* upon brassinolide treatment was carried out as described above for hydroponic culture, with the following modifications. Two-week old *in vitro*-grown plants were transferred to MS medium supplemented with 10 nM 24-epibrassinolide (Sigma-Aldrich, http://www.sigmaaldrich.com/). Seven days later, plants roots were dipped in *V. longisporum* suspension for 30 min and thereafter grown in hydroponic culture supplemented with 10 nM 24-epibrassinolide for the rest of the experiment.

### *In Situ* Detection of H_2_O_2_

H_2_O_2_ was detected in two-week-old *in vitro* grown plants at 2 dpi, by vacuum infiltration of DAB solution for 30 min, followed by a 2 h incubation and subsequent destaining in absolute ethanol [77]. Quantification of DAB was done by measuring the intensity of stained tissues in Adobe Photoshop (Adobe Systems Inc., http://www.adobe.com/). At least three images were taken at 20x magnification on a Zeiss Axioplan microscope, on each of at least five individual plants per genotype and treatment. After conversion of images to black and white, the average intensity of DAB (0–250) was measured on three separate squares (50×50 pixels) for each root image using the histogram function implemented in Photoshop.

### RNA Isolation, cDNA-AFLP and Quantitative Real-time PCR

Total RNA was isolated from *B. oleracea* roots 2 days post VL1 inoculation using Spectrum™ Plant Total RNA Kit (Sigma-Aldrich, http://www.sigmaaldrich.com/). cDNA synthesis, restriction, adapters, primers, amplification steps, transcript profiling and sequencing of selected transcripts were performed as previously described [78]. qRT- PCR on Arabidopsi*s* was performed on materials collected at 2 dpi. RNA isolations and cDNA synthesis were carried out on biological triplicates (roots from at least 25 plants). Each biological replicate was amplified in at least two separate reactions (two technical replicates) in qRT-PCR. Primers were designed using Primer3 [79] and are provided in Table S2. qRT-PCR data were analyzed by the comparative C_T_ method [80] with qRT-PCR efficiency correction determined by the slope of standard curves. Fold-differences in transcript levels and mean standard error were calculated as outlined by Schmittgen and Livak [81].

### Phylogenetic Analyses

Protein sequences from the coding region of all Arabidopsis *RabGAP* genes (http://www.arabidopsis.org) and *B. oleracea* RR86 were aligned using ClustalW (http://www.ebi.ac.uk/Tools/msa/clustalw2/), and manually inspected. One hundred and sixteen conserved amino acids were used in the final analysis. The MEGA 5.1 software [82] suggested the JTT+4G model [83] for sequence evolution, and was used in Maximum likelihood analyses. Five hundred bootstrap replicates were performed.

### Plasmid Construction and Plant Transformation

Promoter sequence for *RabGAP22* was determined using AtcisDB [84]. cDNA or genomic DNA target sequences were PCR amplified using Phusion DNA polymerase (Thermo Scientific, http://www.thermoscientificbio.com/), cloned into suitable donor vectors (Table S3), and introduced to the Gateway system (Invitrogen, http://www.invitrogen.com/) for sequencing. Final *35S_Pro_:RabGAP22*-His, *RabGAP22_Pro_*:*GUS*, *RabGAP22_Pro_:RabGAP22* and *RabGAP22_Pro_:RabGAP22*-*GFP* constructs were introduced to *Agrobacterium tumefaciens* strain C58, and Arabidopsis Col-0 or *rabgap22-1* plants were transformed using floral-dip [85]. Confirmed T2 lines were used for GUS and GFP analyses and T3 homozygous complementation lines for inoculation assays. At least three independent transgenic lines per construct were used for all analyses.

### *GUS* Expression, BiFC Analysis and Confocal Microscopy

Transgenic Col-0 plants harboring *RabGAP22_Pro_:GUS* and *BAK1_Pro_:GUS* [71] were used for GUS staining [86] and images were taken using a Leica Z16 APO microscope. Transient GFP expression of *RabGAP22_Pro_:RabGAP22*-*GFP* was monitored in *Agrobacterium*-infiltrated *Nicotiana benthamiana* leaves. For BiFC analysis [87], *AGT1* and *RabGAP22* cDNAs were amplified by PCR using primers listed in Table S3. PCR products were ligated into the pCRTM8/GX/TOPO entry vector and sequenced, and then transferred into Gateway compatible BiFC vectors (*pSITE-nEGFP-C1* and *pSITE-cEGFP-C1*) by LR reactions. The *pSITE-cEGFP-AGT1* and *pSITE-nEGFP-RabGAP22* plasmids were subsequently transformed into the *Agrobacterium* strain GV3101. For determination of organelle localization, the nuclear marker VirD2NLS-YFP [32] or a peroxisomal-targeted mCherry marker [88] were used in tobacco co-infiltration settings. For co-infiltrations, bacteria harboring the different plasmids were mixed in 1∶1 or 1∶1: 1 (v/v) ratios as appropriate and infiltrated into leaves of two-week-old *N. benthamiana* plants [89]. Fluorescence and brightfield images were taken using a Zeiss 780 confocal scanning microscope at 4 d post infiltration. GFP was excited at 488 nm, and detected at 493–530 nm, YFP excited at 514 nm and detected at 518–560 nm, chlorophyll excited at 633 nm and detected at 647–721 nm, and mCherry excited at 561 nm and detected at 600–650 nm. Images were analyzed using the built in Zen2011 software.

### ABA and JA Analysis

*In vitro* grown Arabidopsis Col-0 and *rabgap22-1* plants inoculated with VL1 or water were harvested at 2 dpi, in biological triplicates consisting of at least 50 whole plants. Endogenous levels of ABA and JA were extracted and quantified using GC-MS [90], [91].

### Stomata Assays

Leaf peels were collected from the abaxial side of mature leaves from four-week-old plants and placed in 300 µl H_2_O or buffer (25 mM MES, 10 mM KCl, pH 6.15) for 30 minutes. For ABA and flg22 treatments, the buffer was supplemented with either 10 µM ABA (Sigma-Aldrich), or 10 µM flg22 (EZBiolab, http://www.ezbiolab.com/). For bacterial assays, a cell suspension of 1·10^8^ *Pst* DC3000 ml^−1^ was sprayed on the leaves, and samples collected at 1 and 4 h post inoculation. Leaves were stained with 20 µM propidium iodide and stomatal measurements carried out according to Chitrakar and Melotto [92]. Leaf peels were observed at a Zeiss Axioplan microscope. The width of the stomatal pore opening was measured at the widest size on at least 60 randomly selected stomata for each genotype and treatment. Stomatal measurements on *V. longisporum* inoculated plants was performed in the same way as for *Pst* DC3000, except samples were taken 14 d post inoculation.

### Protein Extraction, Immunoprecipitation and Mass Spectrometry

Total proteins were extracted from leaves of *35S_Pro_:RabGAP22-*His transgenic Col-0 plants with extraction buffer [50 mM Tris-Hcl pH7.4, 10 mM EDTA, 0.1% Triton X-100 and 1 µl ProteoBlock™ protease inhibitor cocktail (Fermentas, http://www.thermoscientificbio.com/)]. For Western blot, 10 µg of total protein per lane was separated in 12% SDS-PAGE gels and electro-transferred to a PVDF membrane. Primary anti-His antibody (Invitrogen, http://www.invitrogen.com/) and peroxidase-conjugated secondary antibodies (goat antirabbit IgG, rabbit anti-mouse IgG, Dako, http://www.dako.com/) were used and detected by chemiluminescence (Amersham, http://www.gelifesciences.com/). For immune-detection, crude extracts were immune-precipitated with anti-His antibody overnight at 4**°**C. Protein A Sepharose 4 Fast flow beads (GE Healthcare, http://www3.gehealthcare.com/) were added followed by 90 min incubation and rinses with IP washing buffer A [50 mM Tris (pH 8.0), 150 mM NaCl, 0.1% Triton X-100] and IP Washing buffer B [50 mM Tris (pH 8.0), 0.1% Triton X-100]. Two time concentrated SDS sample buffer was added to the final bead-pellets and the precipitated proteins were separated in 12% SDS-PAGE gels followed by colloidal Coomassie staining. MALDI MS/MS analyses of candidate proteins were performed on an Ultraflex III TOF/TOF (Bruker Daltonics, http://www.bruker.com/). Amino acid sequences were identified by comparing their MS/MS spectra against an Arabidopsis subset of the NCBI database, using the Mascot program (http://www.matrixscience.com/).

## Supporting Information

Figure S1 **Arabidopsis** ***RabGAP22*** **is required for defense to** ***Verticillium longisporum.*** (A) Figure showing the locations of T-DNA insertions in *rabgap* mutants. (B) Phenotypes of soil-inoculated Arabidopsis plants. *rabgap22-1* and *rabgap22-2* mutants showed increased susceptibility to the fungus, whereas *rabgap11-1, rabgap19-1* and *rabgap20-1* plants had a disease phenotype similar to Col-0. Images taken 28 days post inoculation. The experiment was repeated three times. (C) Relative transcript levels of *RabGAP11*, *RabGAP19*, *RabGAP20* and *RabGAP22* in their respective T-DNA insertion mutants. Values are means ± SE (>10 plants per genotype). Asterisks indicate significant difference to the respective transcript level in Col-0 (Student’s t-test; *p≤0.05; **p≤0.01; ***p≤0.001).
(TIF)

Figure S2 **Histochemical localization of GUS activity in** ***in vitro*** **-grown** ***RabGAP22_Pro_:GUS*** **transgenic plants at different development stages.** (A) 2 days old seedling, with GUS staining throughout the plant, in particular in the root meristem (B) 5 days old seedling, with strong staining in the vascular tissues (C) 21 days old plant, with staining in the vascular tissues (D) stomata-localized staining in leaf of 7 days old seedling (E) flower, with GUS staining in style and receptacle (F) silique (G) seed pod.
(TIF)

Figure S3 **RabGAP22 interacts with SERINE GLYOXYLATE AMINOTRANSFERASE 1 (AGT1)** ***in planta*** **.** (A) Coomassie stained SDS-PAGE gel, with immunoprecipitated proteins from soil-grown *35S_Pro_:RabGAP22*-His plants. In mock plants, only a ∼60 kDa band corresponding to RabGAP22 was detected, whereas in inoculated plants, a ∼45 kDa band, corresponding to the size of AGT1, co-immunoprecipitated together with RabGAP22. (B) MALDI MS/MS analysis on the co-immunoprecipitated protein. The peptide fragments identify the protein as AGT1. Protein score is −10·Log(P), where P is the probability that the observed match is a random event. Protein scores greater than 86 are significant (p<0.05). (C) ClustalW alignment of AGT1 protein sequences from *V. longisporum* (KF242188), *Magnaporthe oryzae* (MGG_02525.6) and *Arabidopsis thaliana* (At2g13360.1). Peptide fragments identified in the MALDI-MS/MS are highlighted in grey, and are 100% identical to the Arabidopsis sequence. Vl = *V. longisporum*, Mo = *M. oryzae*, At = Arabidopsis. (D) Relative transcript levels of *AGT1* in roots of *in vitro*-grown Arabidopsis plants 2 d post inoculation with *V. longisporum*. Values are means ± SE (n = 3 pools of >20 plants, experiment repeated twice). (E) Relative fungal DNA content in roots of Arabidopsis plants grown in hydroponic culture, quantified at 14 dpi using qRT-PCR. Data represent means ± SE (n = 3 pools of 5 plants). (F, G) Detection of H_2_O_2_ by DAB staining of *V. longisporum* inoculated *in vitro* grown Arabidopsis plants at 2 dpi. (F) Phenotypes at 2 dpi, showing reduced DAB staining intensity in inoculated *rabgap22-1* compared to Col-0. (G) Quantification of DAB staining intensity. (DAB = 3,3′-diaminobenzidine). Asterisks indicate significant difference to the respective Col-0 mock treated control (Student’s t-test; *p≤0.05; **p≤0.01; ***p≤0.001).
(TIF)

Figure S4 ***In silico*** **analysis on** ***RabGAP22*** **using the co-expression analysis tool available at Genevestigator v3.** (A) The 25 genes most correlated to *RabGAP22* expression. (B) Genevestigator *RabGAP22* expression data limited to response to hormone treatments.
(TIF)

Figure S5 **BAK1 contributes to** ***Verticillium longisporum*** **resistance.** Histochemical localization of *GUS* activity in *in vitro*-grown transgenic Arabidopsis plants harboring a *BAK1_Pro_*:*GUS* construct, 1 and 2 d post inoculation with *V. longisporum* or water. Experiment was repeated twice.
(TIF)

Figure S6 **Hypothetical model for RabGAP22 in early defense to** ***Verticillium longisporum*** **.** The RabGTPase activating genes are known to serve as molecular switches in a wide range of pathways. In congruency, we see multiple functions of RabGAP22 in the response to *V. longisporum*. In a pathogen recognition complex (dashed line), with a so far unidentified PAMP molecule and receptor, RabGAP22 could act together with BAK1, most likely interfering with the phosphorylation steps required for BAK1 activation. In response to *V. longisporum* RabGAP22 would then re-localize from the nucleus to the peroxisome, where it interacts with the photorespiratory protein AGT1. In peroxisomal JA biosynthesis, OPDA is stepwise converted to JA, which after release is subsequently conjugated to the bioactive JA-Ile in the cytosol. By a so far unidentified mechanism, RabGAP22/AGT may interfere with the multiple steps leading to formation of JA, thereby also indirectly impacts JA-Ile signaling. As extensive cross-talk takes place between the phytohormones JA and ABA, altered JA levels may also contribute to the impairment in stomatal closure responses seen in the *rabgap22-1* mutants. (AGT1 = SERINE GLYOXYLATE AMINOTRANSFERASE 1; BAK1 = BRI1-ASSOCIATED RECEPTOR KINASE 1; JA = jasmonic acid; JA-Ile = JA-Isoleucine; OPDA = 12-oxo-phytodienoic acid; PAMP = Pathogen-Associated Molecular Pattern).
(TIF)

Table S1 **Transcript derived fragments (TDF) up-regulated in** ***B. oleracea*** **cv. BRA723 at two days post inoculation with** ***V. longisporum*** **.** Locus, function and E values represent the best BLAST hit for each of the TDFs. Loci have been divided into functional categories as determined by GO Molecular Function at TAIR (http://www.arabidopsis.org).
(DOCX)

Table S2 **Primers used for quantitative real-time PCR (qRT-PCR).**
(DOCX)

Table S3 **Primers, vectors, templates and plant genotypes used for transgenic constructs.**
(DOCX)
