# Supplementary figures and images for: RabGAP22 Is Required for Defense to the Vascular Pathogen Verticillium longisporum and Contributes to Stomata Immunity
Source: PLoS One. 2014 Feb 4;9(2):e88187. doi: 10.1371/journal.pone.0088187 (PMC3913773; doi:10.1371/journal.pone.0088187)

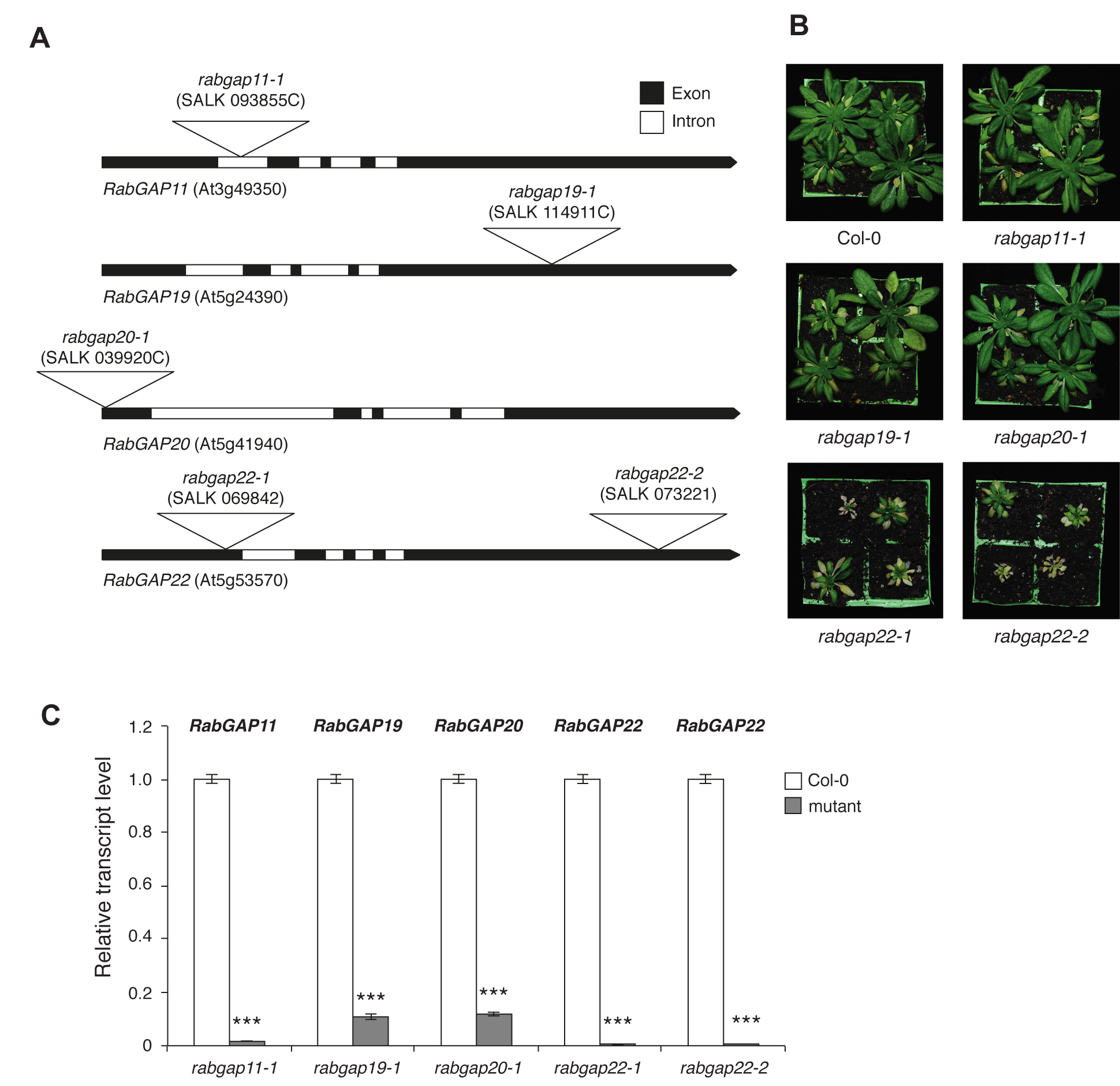

Supplement: Figure S1 — Arabidopsis RabGAP22 is required for defense to Verticillium longisporum. (A) Figure showing the locations of T-DNA insertions in rabgap mutants. (B) Phenotypes of soil-inoculated Arabidopsis plants. rabgap22-1 and rabgap22-2 mutants showed increased susceptibility to the fungus, whereas rabgap11-1, rabgap19-1 and rabgap20-1 plants had a disease phenotype similar to Col-0. Images taken 28 days post inoculation. The experiment was repeated three times. (C) Relative transcript levels of RabGAP11, RabGAP19, RabGAP20 and RabGAP22 in their respective T-DNA insertion mutants. Values are means ± SE (>10 plants per genotype). Asterisks indicate significant difference to the respective transcript level in Col-0 (Student’s t-test; *p≤0.05; **p≤0.01; ***p≤0.001). (TIF) [file pone.0088187.s001.tif]

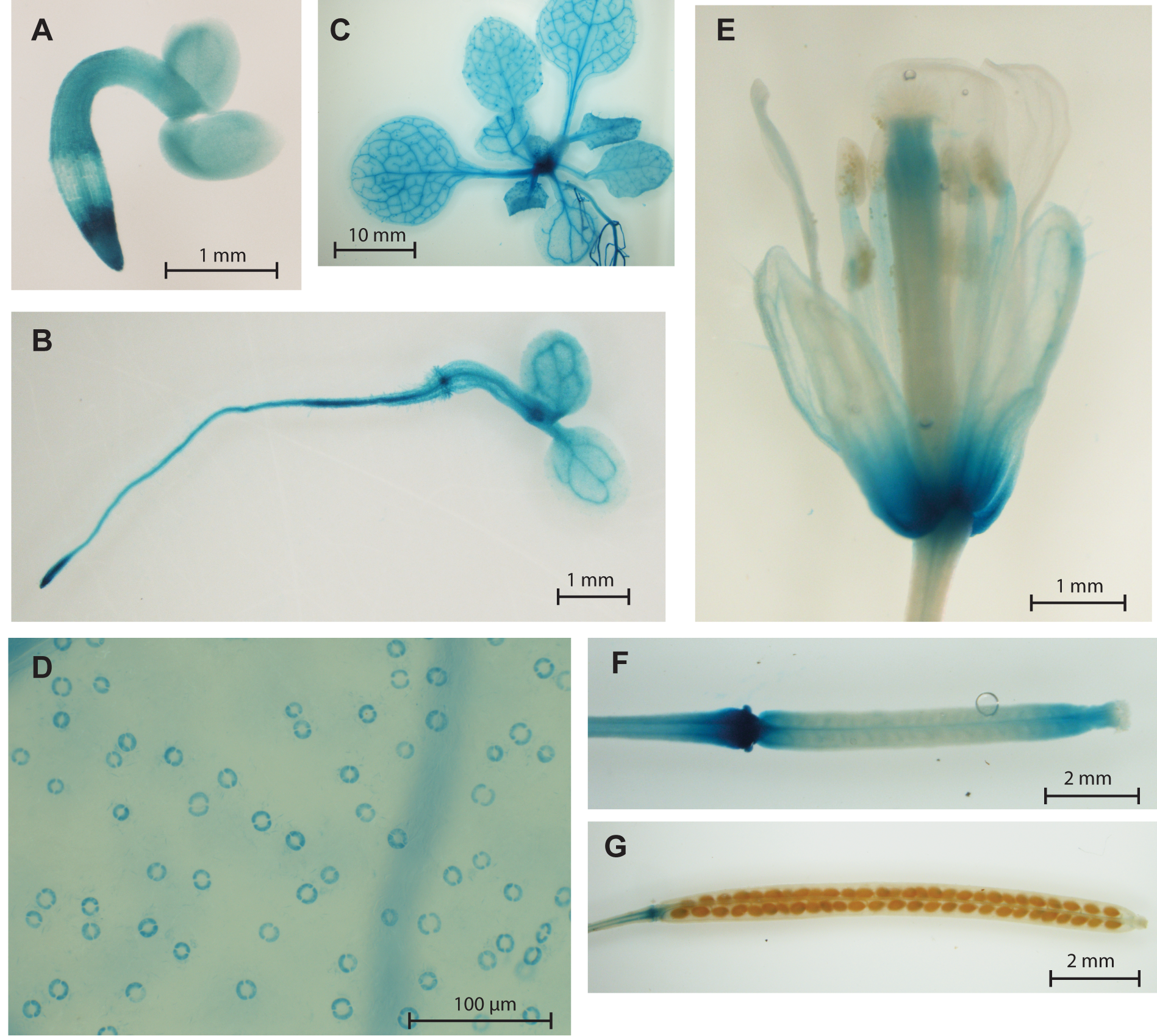

Supplement: Figure S2 — Histochemical localization of GUS activity in in vitro -grown RabGAP22Pro:GUS transgenic plants at different development stages. (A) 2 days old seedling, with GUS staining throughout the plant, in particular in the root meristem (B) 5 days old seedling, with strong staining in the vascular tissues (C) 21 days old plant, with staining in the vascular tissues (D) stomata-localized staining in leaf of 7 days old seedling (E) flower, with GUS staining in style and receptacle (F) silique (G) seed pod. (TIF) [file pone.0088187.s002.tif]

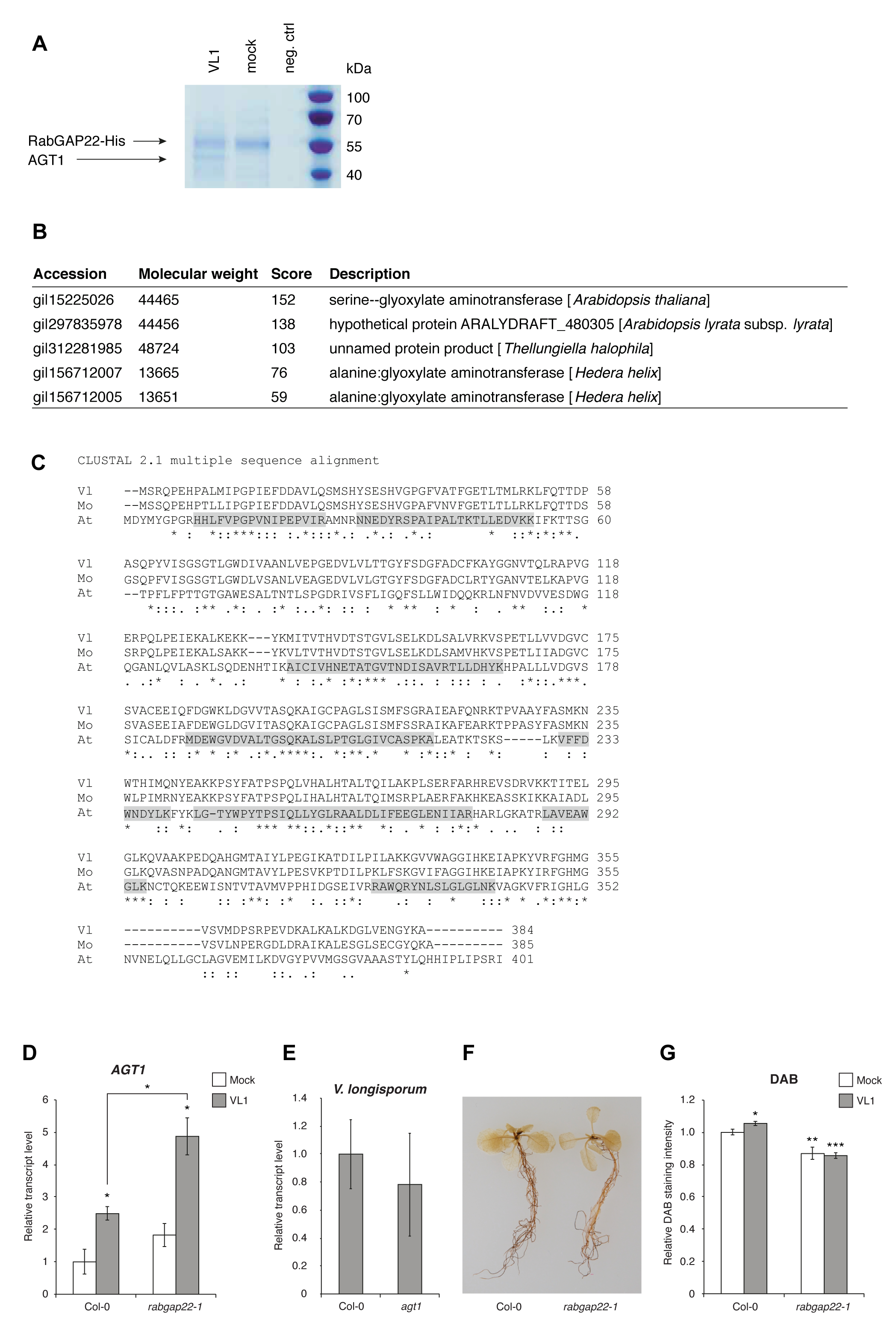

Supplement: Figure S3 — RabGAP22 interacts with SERINE GLYOXYLATE AMINOTRANSFERASE 1 (AGT1) in planta . (A) Coomassie stained SDS-PAGE gel, with immunoprecipitated proteins from soil-grown 35SPro:RabGAP22-His plants. In mock plants, only a ∼60 kDa band corresponding to RabGAP22 was detected, whereas in inoculated plants, a ∼45 kDa band, corresponding to the size of AGT1, co-immunoprecipitated together with RabGAP22. (B) MALDI MS/MS analysis on the co-immunoprecipitated protein. The peptide fragments identify the protein as AGT1. Protein score is −10·Log(P), where P is the probability that the observed match is a random event. Protein scores greater than 86 are significant (p<0.05). (C) ClustalW alignment of AGT1 protein sequences from V. longisporum (KF242188), Magnaporthe oryzae (MGG_02525.6) and Arabidopsis thaliana (At2g13360.1). Peptide fragments identified in the MALDI-MS/MS are highlighted in grey, and are 100% identical to the Arabidopsis sequence. Vl = V. longisporum, Mo = M. oryzae, At = Arabidopsis. (D) Relative transcript levels of AGT1 in roots of in vitro-grown Arabidopsis plants 2 d post inoculation with V. longisporum. Values are means ± SE (n = 3 pools of >20 plants, experiment repeated twice). (E) Relative fungal DNA content in roots of Arabidopsis plants grown in hydroponic culture, quantified at 14 dpi using qRT-PCR. Data represent means ± SE (n = 3 pools of 5 plants). (F, G) Detection of H2O2 by DAB staining of V. longisporum inoculated in vitro grown Arabidopsis plants at 2 dpi. (F) Phenotypes at 2 dpi, showing reduced DAB staining intensity in inoculated rabgap22-1 compared to Col-0. (G) Quantification of DAB staining intensity. (DAB = 3,3′-diaminobenzidine). Asterisks indicate significant difference to the respective Col-0 mock treated control (Student’s t-test; *p≤0.05; **p≤0.01; ***p≤0.001). (TIF) [file pone.0088187.s003.tif]

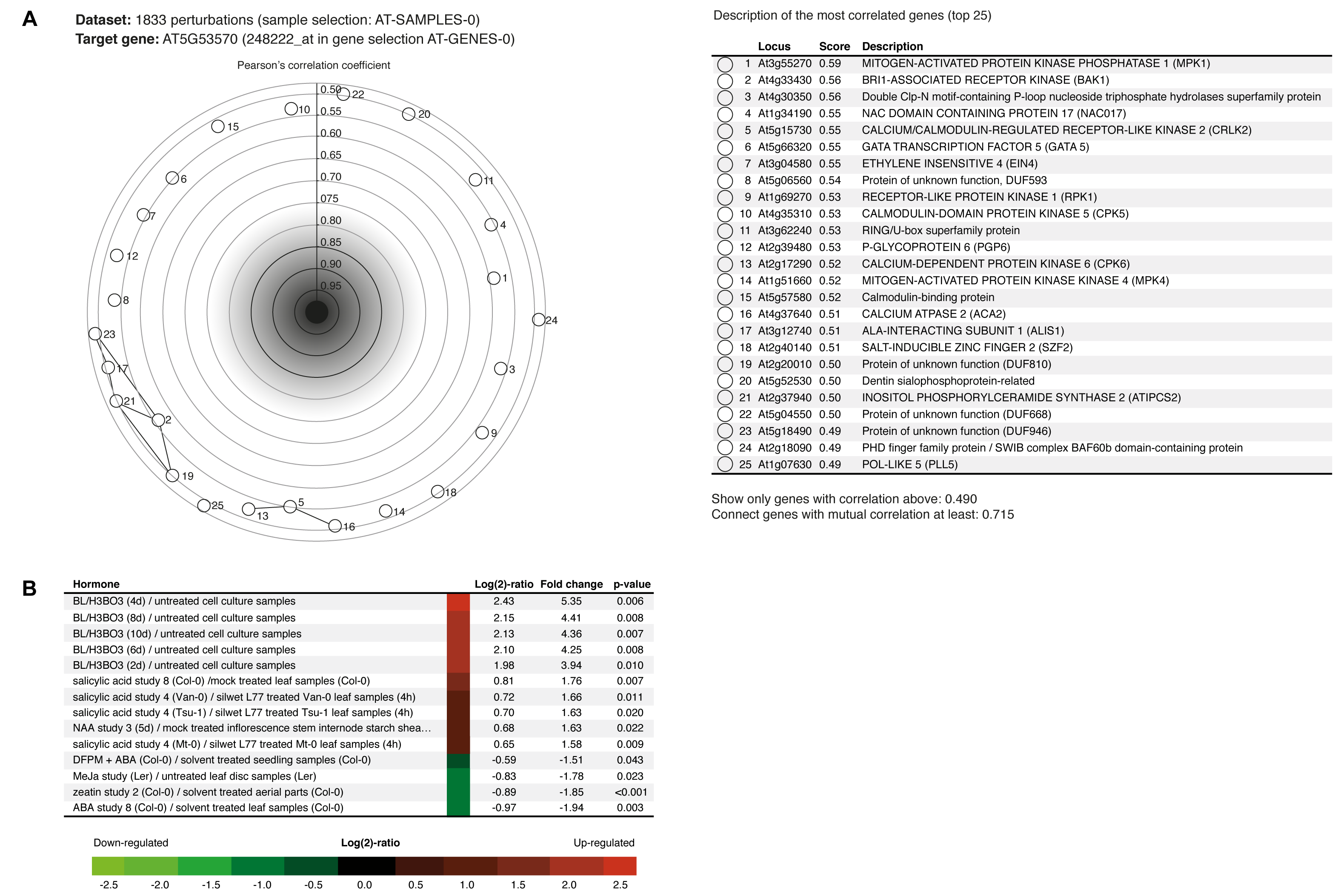

Supplement: Figure S4 — In silico analysis on RabGAP22 using the co-expression analysis tool available at Genevestigator v3. (A) The 25 genes most correlated to RabGAP22 expression. (B) Genevestigator RabGAP22 expression data limited to response to hormone treatments. (TIF) [file pone.0088187.s004.tif]

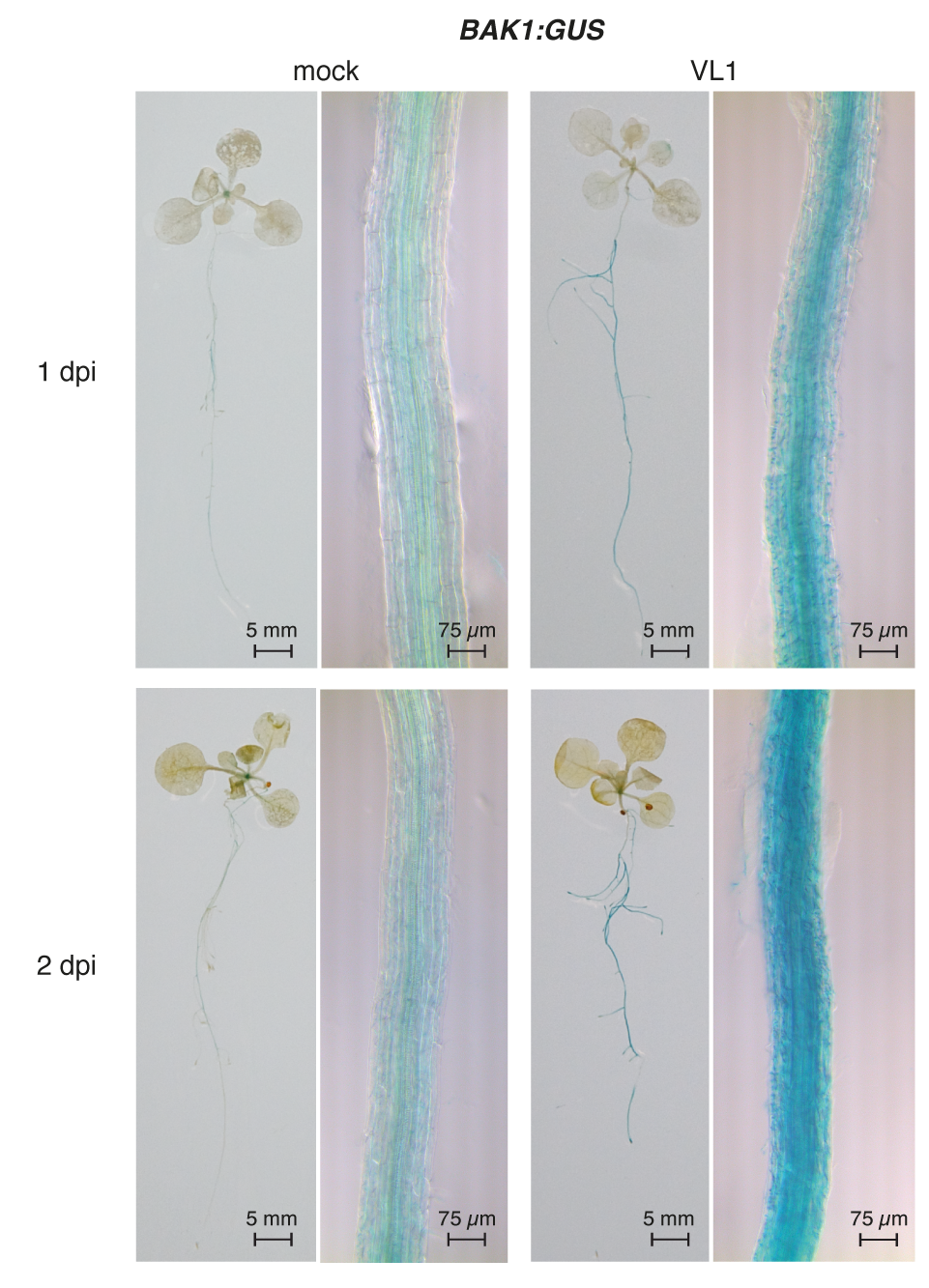

Supplement: Figure S5 — BAK1 contributes to Verticillium longisporum resistance. Histochemical localization of GUS activity in in vitro-grown transgenic Arabidopsis plants harboring a BAK1Pro:GUS construct, 1 and 2 d post inoculation with V. longisporum or water. Experiment was repeated twice. (TIF) [file pone.0088187.s005.tif]

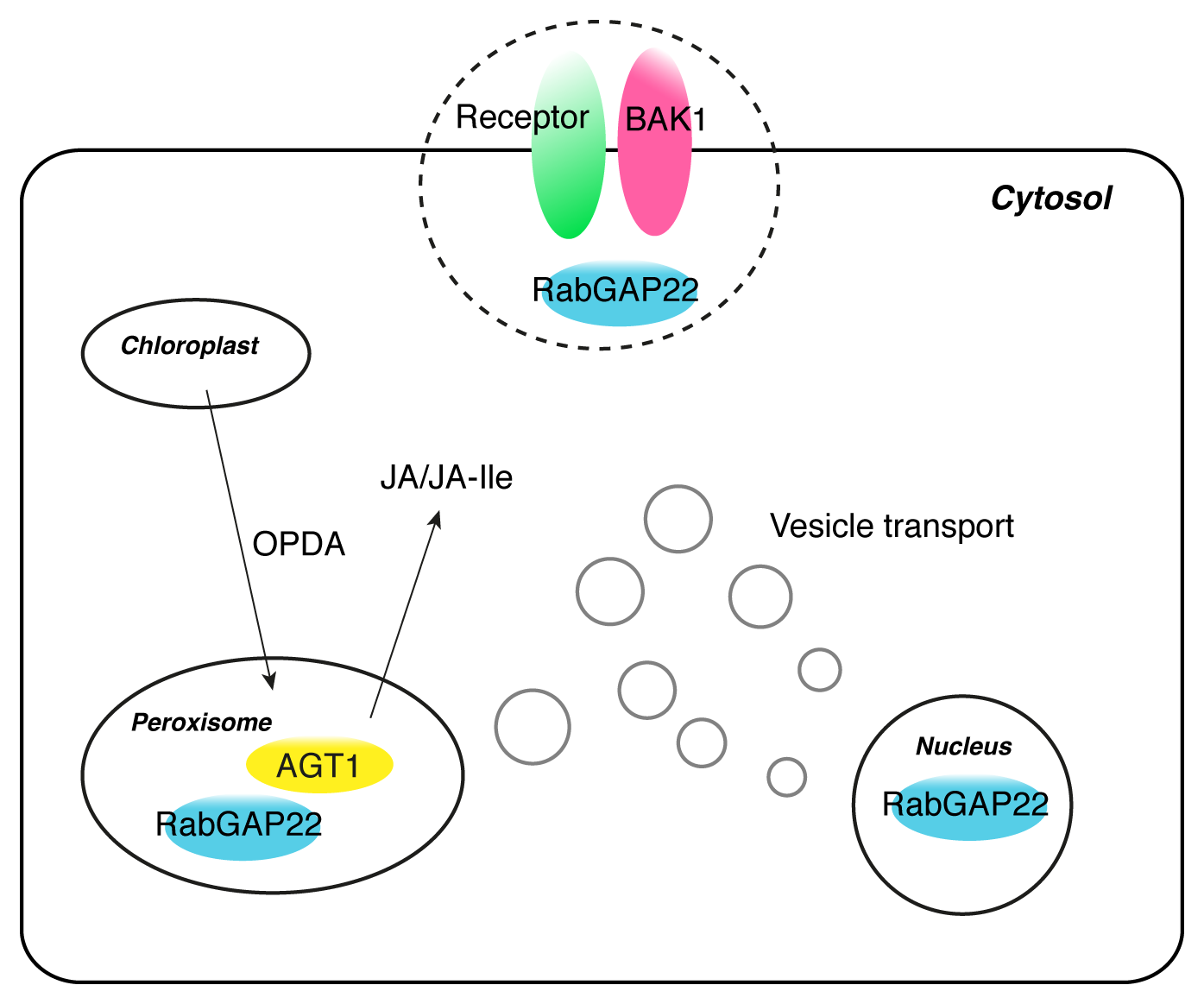

Supplement: Figure S6 — Hypothetical model for RabGAP22 in early defense to Verticillium longisporum . The RabGTPase activating genes are known to serve as molecular switches in a wide range of pathways. In congruency, we see multiple functions of RabGAP22 in the response to V. longisporum. In a pathogen recognition complex (dashed line), with a so far unidentified PAMP molecule and receptor, RabGAP22 could act together with BAK1, most likely interfering with the phosphorylation steps required for BAK1 activation. In response to V. longisporum RabGAP22 would then re-localize from the nucleus to the peroxisome, where it interacts with the photorespiratory protein AGT1. In peroxisomal JA biosynthesis, OPDA is stepwise converted to JA, which after release is subsequently conjugated to the bioactive JA-Ile in the cytosol. By a so far unidentified mechanism, RabGAP22/AGT may interfere with the multiple steps leading to formation of JA, thereby also indirectly impacts JA-Ile signaling. As extensive cross-talk takes place between the phytohormones JA and ABA, altered JA levels may also contribute to the impairment in stomatal closure responses seen in the rabgap22-1 mutants. (AGT1 = SERINE GLYOXYLATE AMINOTRANSFERASE 1; BAK1 = BRI1-ASSOCIATED RECEPTOR KINASE 1; JA = jasmonic acid; JA-Ile = JA-Isoleucine; OPDA = 12-oxo-phytodienoic acid; PAMP = Pathogen-Associated Molecular Pattern). (TIF) [file pone.0088187.s006.tif]
