# Supplementary material for: RabGAP22 Is Required for Defense to the Vascular Pathogen Verticillium longisporum and Contributes to Stomata Immunity
Source: PLoS One. 2014 Feb 4;9(2):e88187. doi: 10.1371/journal.pone.0088187 (PMC3913773; doi:10.1371/journal.pone.0088187)
Supplement: Table S1 — Transcript derived fragments (TDF) up-regulated in B. oleracea cv. BRA723 at two days post inoculation with V. longisporum . Locus, function and E values represent the best BLAST hit for each of the TDFs. Loci have been divided into functional categories as determined by GO Molecular Function at TAIR (http://www.arabidopsis.org). (DOCX) [file pone.0088187.s007.docx]

**Table S1.** Transcript derived fragments (TDF) up-regulated in *Brassica oleracea* accession BRA723 at two days post inoculation with *Verticillium longisporum*.

| **Functional category** | **Locus** | **Function** | **E value** | **TDF** |
| --- | --- | --- | --- | --- |
| *Signal transduction* | At1g22300 | 14-3-3-like protein GF14 epsilon (GRF10) | 2e-102 | RR29 |
|  | At3g12740 | ALA-interacting subunit 1 (ALIS1) | 4e-29 | RR55 |
|  | **At5g53570** | **RabGAP/TBC domain-containing protein** | **7e-71** | **RR86** |
|  | At4g08500 | mitogen-activated protein kinase kinase kinase 1 (MEKK1) | 4e-67 | RR118 |
| *Cell organization and biogenesis* | At3g07880 | SUPERCENTIPEDE1 (SCN1), has Rho GDP-dissociation inhibitor activity | 8e-45 | RR113 |
|  | At5g15920 | STRUCTURAL MAINTENANCE OF CHROMOSOMES 5 (SMC5) | 7e-39 | RR145 |
| *Developmental processes* | At5g09810 | ACTIN 7 | 5e-41 | RR6 |
|  | At2g39770 | CYTOKINESIS DEFECTIVE 1 | 3e-75 | RR62 |
|  | At3g09190 | Putative lectin | 2e-14 | RR102 |
|  | At5g19430 | RING/U-box domain-containing protein | 3e-88 | RR130 |
| *DNA or RNA metabolism* | At5g63420 | RNA-metabolising metallo-beta-lactamase family | 7e-77 | RR68 |
| *Electron transport or energy pathways* | At4g30190 | PLASMA MEMBRANE PROTON ATPASE 2 (PMA2) | 2e-21 | RR38 |
|  | At3g52990 | Pyruvate kinase | 2e-47 | RR54 |
|  | At2g02050 | NADH-ubiquinone oxidoreductase B18 subunit | 2e-15 | RR98 |
|  | At2g27510 | FERREDOXIN 3 (FD3) | 2e-116 | RR103 |
|  | At5g56090 | cytochrome c oxidase subunit XV assembly protein (COX15) | 8e-64 | RR122 |
| *Other biological and cellular processes* | At1g04130 | TETRATRICOPEPTIDE REPEAT 2 (TPR2) | 1e-17 | RR17 |
|  | At5g07440 | glutamate dehydrogenase 2 | 2e-46 | RR31 |
|  | At1g54100 | Aldehyde dehydrogenase 7B4 (ALDH7B4) | 3e-62 | RR47 |
|  | At1g26550 | peptidyl-prolyl cis-trans isomerase PPIC-type family protein | 1e-62 | RR68-2 |
|  | At2g30490 | CINNAMATE 4-HYDROXYLASE (C4H) | 1e-35 | RR111 |
|  | At3g52930 | FRUCTOSE-BISPHOSPHATE ALDOLASE 8 | 4e-86 | RR129-1 |
|  | At5g03300 | ADEONOSINE KINASE PROTEIN 2 (ADK2) | 1e-22 | RR2 |
|  | At3g11520 | CYCLIN B1;3 | 7e-65 | RR8 |
|  | At5g64740 | CELLULOSE SYNTHASE 6 | 4e-42 | RR10 |
|  | At5g65430 | 14-3-3-like protein GF14 kappa (GRF8) | 9e-38 | RR32 |
|  | At1g20610 | cyclin-B2-3 (CYCB2;3) | 8e-26 | RR34 |
|  | At4g34710 | Arginine decarboxylase 2 (ADC2) | 4e-17 | RR44 |
|  | At2g32850 | Putative serine/threonine protein kinase | 1e-100 | RR48 |
|  | At5g17920 | COBALAMIN-INDEPENDENT METHIONINE SYNTHASE (ATMS1) | 7e-46 | RR77 |
|  | At3g51840 | acyl-coenzyme A oxidase 4 (ACX4) | 0 | RR82 |
|  | At2g29460 | glutathione S-transferase (GSTU4) | 4e-112 | RR83 |
|  | At1g02090 | COP9 signalosome complex subunit 7 (FUS5) | 2e-65 | RR88 |
|  | At2g31900 | myosin-like protein XIF (XIF) | 3e-25 | RR112 |
|  | At5g12260 | glycosyltransferase family protein | 8e-102 | RR117 |
|  | At5g42620 | metalloendopeptidase/metallopeptidase/zinc ion binding | 2e-70 | RR120 |
|  | At5g13190 | Uncharacterized protein | 6e-21 | RR126 |
|  | At4g19185 | nodulin MtN21 /EamA-like transporter family protein | 2e-40 | RR131 |
| *Other metabolic processes* | At3g52880 | MONODEHYDROASCORBATE REDUCTASE 1 (MDAR1) | 7e-84 | RR4 |
|  | At3g47040 | Glycosyl hydrolase family protein | 7e-84 | RR27 |
|  | At2g41290 | strictosidine synthase-like 2 (SSL2) | 5e-22 | RR28 |
|  | At5g63910 | prenylcysteine oxidase (FCLY) | 1e-17 | RR39 |
|  | At2g47510 | FUMARASE HYDRATASE 1 (FUM1) | 7e-84 | RR40 |
|  | At5g03630 | monodehydroascorbate reductase (NADH)-like protein | 6e-72 | RR58 |
|  | At4g30140 | GDSL esterase/lipase (CDEF1) | 2e-45 | RR71 |
|  | At5g62790 | 1-DEOXY-D-XYLULOSE 5-PHOSPHATE REDUCTOISOMERASE (DXR) | 4e-48 | RR74-1 |
|  | At3g59970 | METHYLENETETRAHYDROFOLATE REDUCTASE 1, MTHFR1 | 1e-100 | RR74-2 |
|  | At3g55440 | TRIOSEPHOSPHATE ISOMERASE | 1e-41 | RR78 |
|  | At4g29840 | threonine synthase 1 (MTO2) | 2e-97 | RR85 |
|  | At2g18770 | Signal recognition particle receptor, beta subunit | 8e-64 | RR89 |
|  | At1g04410 | malate dehydrogenase | 4e-11 | RR110 |
|  | At5g13200 | GRAM domain family protein | 5e-98 | RR129-2 |
|  | At4g24620 | PGI1 At4g24620 | 7e-52 | RR135-2 |
|  | At2g01720 | Ribophorin I, has oligosaccharyl transferase activity | 4e-118 | RR141 |
|  | At5g39990 | Core-2/I-branching beta-1,6-N-acetylglucosaminyltransferase family protein | 3e-63 | RR143 |
| *Protein metabolism* | At1g26630 | translation initiation factor eIF-5A (FBR12) | 5e-33 | RR9 |
|  | At3g12620 | Protein Phosphatase 2C family protein | 3e-95 | RR13 |
|  | At1g56045 | 60S ribosomal protein L41 | 9e-13 | RR15 |
|  | At5g41340 | ubiquitin-conjugating enzyme E2 4 (UBC4) | 3e-24 | RR19 |
|  | At1g11910 | aspartic proteinase A1 (APA1) | 1e-30 | RR59 |
|  | At3g23390 | 60S ribosomal protein L36a | 3e-19 | RR60 |
|  | At3g13920 | translational initiation factor 4A-1 (EIF4A-1) | 1e-149 | RR104 |
|  | At1g07870 | putative serine/threonine-protein kinase RLCKVII | 3e-107 | RR106 |
|  | At5g38210 | serine/threonine protein kinase family protein At5g38210 | 4e-42 | RR107 |
| *Response to abiotic or biotic stimulus* | At3g22840 | EARLY LIGHT-INDUCIBLE PROTEIN 1 (ELIP1) | 3e-82 | RR1 |
|  | At4g13850 | glycine-rich RNA-binding protein 2 (GR-RBP2) | 9e-13 | RR24 |
|  | At2g39760 | MATH-BTB domain-containing protein (BPM3) | 2e-08 | RR57 |
|  | At1g76180 | dehydrin ERD14 (ERD14), response to dehydration | 1e-73 | RR84 |
| *Response to stress* | At4g19530 | TIR-NB-LRR disease resistance protein | 5e-22 | RR3 |
|  | At4g39260 | COLD, CIRCADIAN RHYTHM, AND RNA BINDING 1 (CCR1) | 5e-35 | RR14 |
|  | At3g06010 | Homeotic gene regulator (ATCHR12) | 9e-95 | RR20 |
|  | At3g01070 | early nodulin-like protein 16 (ENODL16) | 2e-66 | RR49 |
|  | At5g25890 | INDOLE-3-ACETIC ACID INDUCIBLE 28 | 7e-39 | RR99 |
| *Transcription, DNA-dependent* | At2g18160 | BASIC LEUCINE-ZIPPER 2 | 9e-19 | RR18 |
|  | At1g01060 | LATE ELONGATED HYPOCOTYL 1 (LHY1) | 2e-89 | RR50 |
|  | At5g65790 | MYB domain protein 68 (MYB68) | 2e-27 | RR125 |
| *Transport* | At4g39220 | ATRER1A, Key player of retrieval of ER membrane proteins | 4e-29 | RR11 |
|  | At3g16270 | ENTH/VHS family protein | 4e-67 | RR33 |
|  | At4g15160 | Bifunctional inhibitor/lipid-transfer protein/seed storage 2S albumin superfamily protein | 1e-22 | RR41 |
|  | At4g11790 | Ran-binding protein 1 domain containing protein | 2e-47 | RR43 |
|  | At3g30390 | amino acid transporter protein, At3g30390 | 3e-88 | RR51 |
|  | At1g55840 | Sec14p-like phosphatidylinositol transfer family protein | 4e-29 | RR56 |
|  | At4g30420 | nodulin MtN21 /EamA-like transporter family protein | 2e-46 | RR70 |
|  | At3g49940 | LOB DOMAIN-CONTAINING PROTEIN 38 | 3e-24 | RR76 |
|  | At3g20660 | ORGANIC CATION/CARNITINE TRANSPORTER 4 | 1e-79 | RR95 |
|  | At2g38330 | MATE efflux family protein At2g38330 | 7e-52 | RR108 |
|  | At1g75630 | VACUOLAR H+-PUMPING ATPASE C1 (VHA-C1) | 3e-126 | RR115 |
|  | At1g59870 | PDR8 ABC transporter | 2e-32 | RR124 |
|  | At2g18960 | OPEN STOMATA 2 (OST2), PLASMA MEMBRANE PROTON ATPASE (PMA) | 1e-87 | RR142 |
|  | At3g47420 | phosphate starvation-induced protein (PS3) | 6e-40 | RR144 |
| *Unknown biological processes* | At2g41760 | Uncharacterized protein | 3e-37 | RR16 |
|  | At1g07750 | cupin domain-containing protein | 2e-28 | RR21 |
|  | At2g17030 | F-box protein SKIP23 | 2e-14 | RR35 |
|  | At1g07040 | Uncharacterized protein | 2e-21 | RR36 |
|  | At4g31040 | CemA-like proton extrusion protein-like protein | 1e-112 | RR42-2 |
|  | At3g61360 | Pentatricopeptide repeat (PPR) containing protein | 6e-15 | RR46 |
|  | At2g33400 | Uncharacterized protein | 4e-55 | RR52 |
|  | At1g67060 | Uncharacterized protein | 8e-07 | RR92 |
|  | At2g15270 | Uncharacterized protein | 9e-89 | RR96 |
|  | At3g08630 | Protein of unknown function | 2e-34 | RR97 |
|  | At5g61040 | Uncharacterized protein | 4e-10 | RR119 |
|  | At5g44710 | Uncharacterized protein | 5e-47 | RR121 |
|  | At1g15860 | Uncharacterized protein | 1e-22 | RR127 |
|  | At2g33570 | Uncharacterized protein | 1e-43 | RR132 |
| Locus, function and E values represent the best BLAST hit for each of the TDFs. Loci have been divided into functional categories as determined by GO Molecular Function at TAIR (http://www.arabidopsis.org). | | | | |
