# Supplementary material for: RabGAP22 Is Required for Defense to the Vascular Pathogen Verticillium longisporum and Contributes to Stomata Immunity
Source: PLoS One. 2014 Feb 4;9(2):e88187. doi: 10.1371/journal.pone.0088187 (PMC3913773; doi:10.1371/journal.pone.0088187)
Supplement: Table S2 — Primers used for quantitative real-time PCR (qRT-PCR). (DOCX) [file pone.0088187.s008.docx]

**Table S2.** Primers used for quantitative real-time PCR (qRT-PCR).

| **Purpose** | **Primer name** | **Sequence (5'-3')** | **Source** |
| --- | --- | --- | --- |
| *Pathogen quantification* | OLG70 | CAGCGAAACGCGATATGTAG | [1] |
|  | OLG71 | GGCTTGTAGGGGGTTTAGA | [1] |
|  | ACT_F1 | GTATGCTCTTCCTCATGCTATCCTT | [1] |
|  | ACT_R2 | TTCCCGTTCTGCGGTAGTG | [1] |
|  | Ps-F | AAGCAACGCGAAGAACCTTA | This study |
|  | Ps-R | CATGCAGCACCTGTCTCAAT | This study |
|  | UBQ5-F | CGATGGATCTGGAAAGGTTC | This study |
|  | UBQ5-R | AGCTCCACAGGTTGCGTTAG | This study |
| *Gene expression* | ACT_F1 | GTATGCTCTTCCTCATGCTATCCTT | [2] |
|  | ACT_R2 | TTCCCGTTCTGCGGTAGTG | [2] |
|  | RabGAP11-F | GCACCTCCCACTGATGATTT | This study |
|  | RabGAP11-R | AACTGCCCTGCCATACTTTG | This study |
|  | RabGAP19-F | GCTGCTTCTGTGTTGCAAAG | This study |
|  | RabGAP19-R | GGCGTGGAGGGTAACAATAA | This study |
|  | RabGAP20-F | GCACCACCGACAGAAGATTT | This study |
|  | RabGAP20-R | CAGCCATGCTGTTACATTCC | This study |
|  | RabGAP22-F | ACGGATGAGTCTGAAACTGTCAT | This study |
|  | RabGAP22-R | GCTTGATAAACAAGTGAGAAACTGGA | This study |
|  | PDF1.2-F | TGGTGGAAGCACAGAAGTTG | This study |
|  | PDF1.2-F | GATCCATGTTTGGCTCCTTC | This study |
|  | JAZ10-qPCR-F | ATCCCGATTTCTCCGGTCCA | This study |
|  | JAZ10-qPCR-R | ACTTTCTCCTTGCGATGGGAAGA | This study |
|  | COI1-qPCR-F | CATGGCGGTGTATGTCTCAGA | [3] |
|  | COI1-qPCR-R | TCGAGTAAGACAAGGCGGAAGT | [3] |
|  | BAK1-qPCR-F | GGAATCAGAACTCTATCCTTGTGC | This study |
|  | BAK1-qPCR-R | TTTGAGAGATCCAGAACTTGTAGC | This study |
|  | VSP2-qPCR-F1 | GTTAGGGACCOGAGCATCAA | This study |
|  | VSP2-qPCR-R1 | AACGGTCACTGAGTATGATGGGT | This study |
|  | AGT1-qPCR-F | GGCTGAAAAACTGCACACAG | This study |
|  | AGT1-qPCR-R | ATATGCGGAGGCACCATAAC | This study |

1. Eynck C, Koopmann B, Grunewaldt-Stoecker G, Karlovsky P, Tiedemann A (2007) Differential interactions of *Verticillium longisporum* and *V. dahliae* with *Brassica napus* detected with molecular and histological techniques. European Journal of Plant Pathology 118: 259–274.
2. Sohlberg JJ, Myrenås M, Kuusk S, Lagercrantz U, Kowalczyk M, et al. (2006) STY1 regulates auxin homeostasis and affects apical-basal patterning of the Arabidopsis gynoecium. The Plant Journal 47: 112–123.
3. Maruta T, Inoue T, Tamoi M, Yabuta Y, Yoshimura K, et al. (2011) Arabidopsis NADPH oxidases, AtrbohD and AtrbohF, are essential for jasmonic acid-induced expression of genes regulated by MYC2 transcription factor. Plant Science 180: 655–660.
