# Supplementary material for: RabGAP22 Is Required for Defense to the Vascular Pathogen Verticillium longisporum and Contributes to Stomata Immunity
Source: PLoS One. 2014 Feb 4;9(2):e88187. doi: 10.1371/journal.pone.0088187 (PMC3913773; doi:10.1371/journal.pone.0088187)
Supplement: Table S3 — Primers, vectors, templates and plant genotypes used for transgenic constructs. (DOCX) [file pone.0088187.s009.docx]

**Table S3.** Primers, vectors, templates and plant genotypes used for transgenic constructs.

| **Construct** | **Background** | **PCR template** | **Primer (5’-3’)** | **Donor vector** | **Destination vector** |
| --- | --- | --- | --- | --- | --- |
| *RabGAP22:RabGAP22* | *rabgap22-1* | P1 clone MNC6 | F: GGGGACAAGTTTGTACAAAAAAGCAGGCTTTTTTGTTACTGGTCTAAAGATTC | pDONR-Zeo™ | pGWB501^a^ |
|  |  |  | R: GGGGACCACTTTGTACAAGAAAGCTGGGTTGCTTGATAAAGAAGTGAGAAACTG |  |  |
| 35S:*RabGAP22*-GFP | Col-0 | Col-0 cDNA | F: GGGGACAAGTTTGTACAAAAAAGCAGGCTTTGCAAAGTGGTTAGAGAGGCTTC | pDONR-Zeo™ | pGWB551^a^ |
|  |  |  | R: GGGGACCACTTTGTACAAGAAAGCTGGGTTAATACTCTGTGATTGAGAAAGAGTT |  |  |
| 35S:*RabGAP22*-His | Col-0 | Col-0 cDNA | F. CTTAAACAACTCATGCTTGAT | pCR™2.1-TOPO® TA Vector | pGWB8^b^ |
|  |  |  | R: CTGTGATTGAGAAAGAGTTTC |  |  |
| *RabGAP22*:GUS | Col-0 | Col-0 cDNA | F: GGGGACAAGTTTGTACAAAAAAGCAGGCTTTTTTGTTACTGGTCTAAAGATTC | pDONR-Zeo™ | pGWB533^a^ |
|  |  |  | R: GGGGACCACTTTGTACAAGAAAGCTGGGTTGCTCTTTCTTTTTCAGCTCTT |  |  |
| *RabGAP22*_:_*RabGAP22*-His | Col-0 | Col-0 cDNA | F: CCACTGGTCCACGGTAAAGT | pCR™2.1-TOPO® TA Vector | pGWB7^b^ |
|  |  |  | R: CTGTGATTGAGAAAGAGTTTC |  |  |
| *RabGAP22:RabGAP22*-GFP | Col-0 | Col-0 cDNA | F: CCACTGGTCCACGGTAAAGT | pCR™2.1-TOPO® TA Vector | pGWB4^b^ |
|  |  |  | R: CTGTGATTGAGAAAGAGTTTC |  |  |
| *RabGAP22*-BiFC | Col-0 | Col-0 cDNA | F: ATGCTTGATTTCTGCTACGG | pCR8/GW/TOPO | pSITE-cEGFP^c^ |
|  |  |  | R: GAAAGAGTTTCAATCTTGTCGTGA |  |  |
| AGT1-BiFC | Col-0 | Col-0 cDNA | F: CATAATGGACTATATGTATGGACCAGGG | pCR8/GW/TOPO | pSITE-nEGFP^c^ |
|  |  |  | R: GATTCTAGAGGGAATGAGAGGAA |  |  |

^a^ Nakagawa T, Suzuki T, Murata S, Nakamura S, Hino T, et al. (2007) Improved Gateway binary vectors: high-performance vectors for creation of fusion constructs in transgenic analysis of plants. Biosci, Biotechnol Agrochem 71: 2095–2100.

^b^ Nakagawa T, Kurose T, Hino T, Tanaka K, Kawamukai M, et al. (2007) Development of series of gateway binary vectors, pGWBs, for realizing efficient construction of fusion genes for plant transformation. J Biosci Bioeng 104: 34–41.

^c^ Chakrabarty R, Banerjee R, Chung S-M, Farman M, Citovsky V, et al. (2007) pSITE vectors for stable integration or transient expression of autofluorescent protein fusions in plants: probing *Nicotiana benthamiana*-virus interactions. Mol Plant Microbe Interact 20: 740–750.
